# Deciphering the Role and Mechanism of Decidual Monocyte‐Derived Macrophage Infiltration in Obstetric Antiphospholipid Syndrome at Single‐Cell Resolution

**DOI:** 10.1002/advs.202503480

**Published:** 2025-08-07

**Authors:** Rui Gao, Pingying Qing, Hanxiao Chen, Zhengyan Hu, Qiaoran Yang, Chenyang Lu, Huimin Liu, Rujun Zeng, Yuanting Tang, Fan Yu, Jinbiao Han, Xin Liao, Xun Zeng, Lang Qin

**Affiliations:** ^1^ Reproductive Medical Center, Department of Obstetrics and Gynecology West China Second University Hospital, Sichuan University Chengdu 610041 China; ^2^ Key Laboratory of Birth Defects and Related Diseases of Women and Children, Ministry of Education West China Second University Hospital, Sichuan University Chengdu 610041 China; ^3^ Department of Rheumatology and Immunology, West China Hospital Sichuan University Chengdu 610041 China; ^4^ Department of Obstetrics and Gynecology, West China Second University Hospital Sichuan University Chengdu 610041 China; ^5^ Key Laboratory of Drug‐Targeting and Drug Delivery System of the Education Ministry, Sichuan Engineering Laboratory for Plant‐Sourced Drug and Sichuan Research Center for Drug Precision Industrial Technology West China School of Pharmacy Sichuan University Chengdu 610064 China; ^6^ Division of Rheumatology, Department of Internal Medicine, The Third Affiliated Hospital Sun Yet‐Sen University Guangzhou 510665 China; ^7^ Department of Laboratory Medicine, West China Second University Hospital Sichuan University Chengdu 610041 China; ^8^ Department of the Central Operating Unit, West China Second University Hospital/West China School of Nursing Sichuan University Chengdu 610041 China; ^9^ Development and Related Diseases of Women and Children Key Laboratory of Sichuan Province, West China Second University Hospital Sichuan University Chengdu 610041 China

**Keywords:** antiphospholipid antibody, decidual immune microenvironment, decidual macrophage, obstetric antiphospholipid syndrome, peripheral immune microenvironment

## Abstract

Obstetric antiphospholipid syndrome (OAPS) is an autoimmune disorder characterized by pathologic pregnancies and the presence of antiphospholipid antibodies (aPLs). Despite significant infiltration of decidual macrophages observed in OAPS patients, the underlying connections between decidual and peripheral immune cells remain unclear. In this study, an integrated single‐cell atlas is constructed of the decidua and peripheral blood mononuclear cells (PBMCs) from OAPS patients and HCs. Using this atlas, substantial disparities are identified in immune cells between the decidua and PBMCs. The functional changes in immune cells of OAPS patients are also different in decidua and PBMCs. Moreover, increased infiltration of monocyte‐derived macrophages (MDMs) into the decidua is found to contribute to inflammation and trophoblast dysfunction in OAPS. The role of CCL2 is further discovered in recruiting MDMs, driven by excess CCL2 secreted from decidual macrophages stimulated by the aPLs and β_2_‐glycoprotein I complex via the TLR4‐NF‐κB pathway. Decidual vascular endothelial cells express higher levels of ACKR1, which aggregates CCL2 on their surface. Targeting CCR2 and TLR4 improved pregnancy outcomes in OAPS mouse models induced by aPLs, suggesting that these pathways may serve as potential therapeutic targets for OAPS. This study provides new insights into the pathogenesis of OAPS, particularly regarding decidual MDMs infiltration.

## Graphical abstract

This study uncovers a novel mechanism and therapeutic targets in obstetric antiphospholipid syndrome (OAPS). Antiphospholipid antibody/ß2‐glycoprotein I complex boosts CCL2 and TNF‐α expression in decidual macrophages via TLR4‐NF‐κB. CCL2 accumulates on endothelial surfaces via ACKR1, recruiting monocyte‐derived CCR2^+^ macrophages, driving inflammation, and impairing trophoblast function. Targeting TLR4 and CCR2 may benefit OAPS treatment.

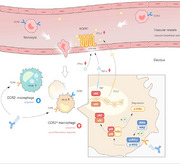

## 1 Introduction

Obstetric antiphospholipid syndrome (OAPS), a subset of antiphospholipid syndrome (APS), is an autoimmune disorder characterized by persistent positive antiphospholipid antibodies (aPLs) in circulation and pathologic pregnancies, including recurrent miscarriage, stillbirth, severe pre‐eclampsia, and severe placental insufficiency.^[^ ^1^ ^]^ Microthrombosis in the maternal‐fetal interface was previously regarded as the primary pathogenesis of OAPS, but subsequent studies found that thrombotic lesions were not common in the placenta of patients.^[^ ^2^ ^]^ The decidual immune cells play essential roles in establishing and maintaining pregnancy. As reported, decidual natural killer cells (NK), macrophages, and T cells interact closely with trophoblasts to maintain tolerance phenotypes and promote embryo development.^[^ ^3^, ^4^ ^]^ Dysfunctions of decidual immune cells have been shown to be associated with pathologic pregnancies such as recurrent miscarriages.^[^ ^5^, ^6^ ^]^ In previous research, we identified significant decidual immune dysfunctions in OAPS patients who suffered recurrent miscarriage by single‐cell transcriptome sequencing (scRNA‐seq).^[^ ^7^ ^]^ Specifically, we found and validated the significant macrophage infiltration in the decidua, which may represent a crucial characteristic and a novel pathogenesis of OAPS.^[^ ^7^ ^]^

However, the origins and regulatory mechanisms of macrophage infiltration in the OAPS decidua, as well as the pathogenic effects of these infiltrating decidual macrophages on trophoblasts, remained unclear, thereby limiting our understanding of the pathogenesis of OAPS. Peripheral monocytes are one of the most important sources of tissue macrophages, and tissue monocyte‐derived macrophages (MDMs) has been reported to contribute to tissue damage in many diseases.^[^ ^8^ ^]^ For instance, in myocardial ischemic injury, damaged tissues recruit peripheral monocytes through the release of specific chemokines.^[^ ^9^ ^]^ MDMs infiltration into the heart has been shown to participate in inflammation and myocardial dysfunction, ihibiting monocytes/macrophages infiltration improved the prognosis of these patients.^[^ ^10^, ^11^ ^]^ Currently, we still have a limited understanding on the peripheral and decidual immune connections in pregnancies and pregnancy complications. Peripheral immune cells undergo functional changes during pregnancy,^[^ ^12^, ^13^ ^]^ and peripheral immune imbalances have been identified in patients experiencing failed pregnancies.^[^ ^14^ ^]^ However, whether and how the peripheral immune cells participate in the occurrence of OAPS, and whether peripheral monocytes are the source of infiltrated decidual macrophages in OAPS patients, all these questions are necessary to be further explored.

To address the aforementioned questions, we constructed an integrated single‐cell atlas of the decidua and peripheral blood mononuclear cells (PBMCs) for OAPS patients and healthy controls (HCs), aiming to systematically elucidate the functions of peripheral and decidual immune cells in OAPS. More importantly, we identified the source of infiltrated decidual macrophages in OAPS and elucidated the functions and detailed mechanisms of MDMs infiltration, providing valuable insights for future therapeutic strategies.

## 2 Results

### 2.1 Integrated Single‐Cell Atlas of Decidua and PBMCs

**Figure 1:**
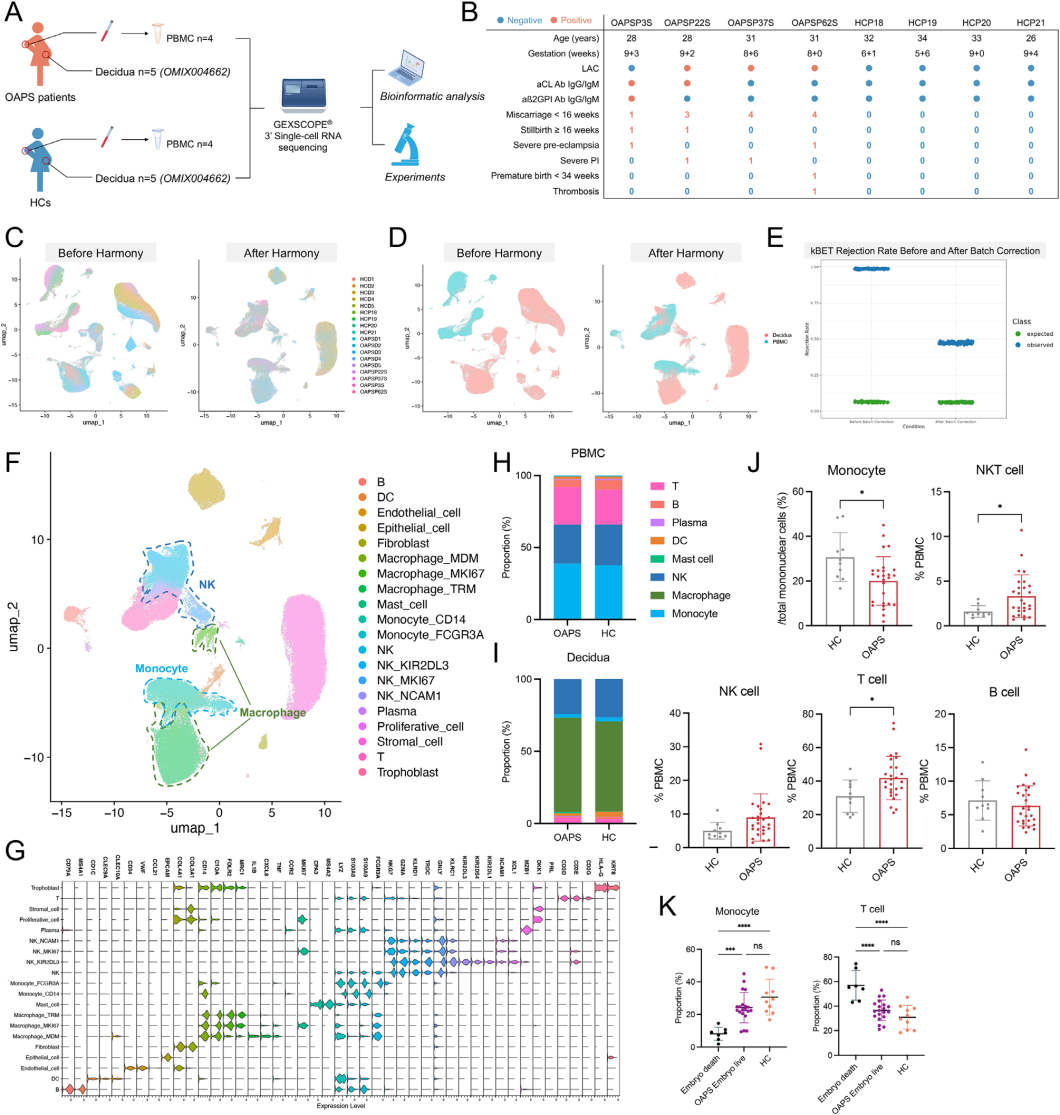
Integrated single‐cell atlas of decidua and PBMCs in OAPS patients and HCs. A) Flowchart depicting the overall design of this study. B) Clinical information of OAPS patients and HCs whose PBMCs were used for scRNA‐seq. C) UMAP plot showing cells from different samples before and after batch effect correction. Different colors represent different samples. D) UMAP plot showing cells from different tissue types before and after batch effect correction. Different colors represent decidua or PBMCs. E) kBET rejection rate before and after batch effect correction. F) UMAP plot of decidual and PBMC cells, indicating 20 cell types. Different colors show different cell types. G) Violin plot showing the canonical marker genes of different cell types. H) Bar plot showing the proportions of identified immune cells in PBMCs of OAPS patients and HCs. I) Bar plot showing the proportions of identified immune cells in the decidua of OAPS patients and HCs. J) FCM analyses of proportions of monocytes, NKT cells, NK cells, T cells, and B cells in PBMCs from HCs (n = 10) and OAPS patients (n = 27). K) Bar plot showing the proportions of monocytes and T cells in PBMCs from OAPS patients with embryo demise or embryo live. Data in (J) are presented as mean ± SD and were analyzed by Student's *t*‐test. Data in (K) are presented as mean ± SD and analyzed using one‐way ANOVA with Tukey's multiple comparisons test. ns, no significant, ^*^ *p* < 0.05, ^**^ *p* < 0.01, ^***^ *p* < 0.001, ^****^ *p* < 0.0001. OAPS, obstetric antiphospholipid syndrome; HCs, healthy controls; PBMC, peripheral blood mononuclear cells; LAC, lupus coagulant; aCL, anticardiolipin antibody; aβ2GPI: anti β2 glycoprotein I antibody; Ab, antibody; scRNA‐seq, single‐cell RNA sequencing; Ig, immunoglobin; PI, placental insufficiency; kBET, k‐Batch Effect Test; NK, natural killer cells; NKT, natural killer T cells; FCM, flow cytometry; DEGs, differential expressed genes; SD, standard deviation; ANOVA, analysis of variance.

PBMCs were collected from four OAPS patients diagnosed with embryo demise and four age‐ and gestational week‐matched HCs during the first trimester of gestation for scRNA‐seq. Additionally, scRNA‐seq data from the decidua of five OAPS patients with embryo demise and five HCs were obtained from a previously published dataset.^[^ ^7^ ^]^ These datasets were integrated to create a comprehensive atlas (**Figure** **1**A). The results of quality control are shown in Figure S1 (Supporting Information). Demographic and clinical information for participants in the scRNA‐seq cohort are summarized in Figure 1B. We also established larger validation cohorts comprising PBMCs and decidua from OAPS patients and HCs to validate the scRNA‐seq findings. Demographic and clinical information for participants in the validation cohorts are presented in Tables S1 and S2 (Supporting Information).

Batch effects were observed between different samples. To address this issue, the Harmony algorithm was used to correct batch effects.^[^ ^15^ ^]^ Following correction, cells from different samples were effectively integrated and mixed (Figure 1C,D). The k‐Batch Effect Test (kBET) metric was used to evaluate the batch correction results.^[^ ^16^ ^]^ After Harmony, the observed kBET rejection rate significantly decreased, indicating successful mitigation of batch effects (Figure 1E). In the integrated atlas, 29 cell clusters were identified and annotated as 20 cell types based on their marker genes: B cells (*CD79A*), dendritic cells (DCs; *CD1C, CLEC9A*), endothelial cells (*CD34*), epithelial cells (*EPCAM*), fibroblasts (*COL4A1*), three types of macrophages (*CD14, C1QA*), mast cells (*CPA3*), two types of monocytes (*LYZ* or *FCGR3A*), four types of NK (*NKG7*, *GZMA*), plasma cells (*MZB1*), proliferative cells (*MKI67*), stromal cells (*DKK1*), T cells (*CD3G*, *CD3D*) and trophoblasts (*HLA‐G*). UMAP plots of cell types and clusters are shown in Figure 1F and Figure S2A (Supporting Information), respectively. Marker genes for each cell type are presented in Figure 1G and Figure S2B (Supporting Information). Numbers of identified cell types are shown in Figure S2C (Supporting Information).

### 2.2 Proportions of Immune Cells Between OAPS Patients and HCs

We first compared the proportions of immune cells between OAPS patients and HCs, both in decidua and PBMCs. Single‐cell data showed similar proportions of PBMC immune cells in OAPS patients and HCs (Figure 1H). However, in decidual tissues, the proportion of macrophages among immune cells tended to increase, while the proportion of NK cells among immune cells tended to decrease (Figure 1I). These findings were validated previously.^[^ ^7^ ^]^ Subsequently, the proportions of immune cells within PBMCs were compared between OAPS patients and HCs in an expanded validation cohort. Our analysis revealed a significant decrease in the proportion of monocytes in OAPS patients, while the proportions of NKT cells and T cells were significantly elevated. Conversely, no statistically significant differences were observed in the proportions of NK cells and B cells (Figure 1J). In the validation cohort, some OAPS patients had experienced embryonic demise, while others remained pregnant during sample collection. In OAPS patients with embryonic demise compared to those with ongoing pregnancies, the proportion of monocytes in PBMCs was lower, while the proportion of T cells was higher (Figure 1K). Gating strategy is shown in Figure S3A (Supporting Information). This finding suggests a potential association between peripheral immune cell changes and miscarriage for OAPS patients.

### 2.3 Functional Differences in Immune Cells from Decidua and PBMCs

We examined functional differences between decidual and PBMCs immune cells. Myeloid cells (monocytes and DCs) from PBMCs expressed elevated levels of genes related to protein synthesis and immune defense. In contrast, myeloid cells (macrophages and DCs) from the decidua expressed elevated levels of genes related to angiogenesis, growth support, and chemotaxis (Figure S4A, Supporting Information). These findings align with prior studies demonstrating that decidual macrophages interact with trophoblasts to promote blood vessel development and maintain immune homeostasis.^[^ ^17^, ^18^ ^]^ NK cells from PBMCs exhibited higher expression of genes associated with cytotoxicity and inflammation, while decidual NK cells exhibited higher expression of genes associated with inhibitory receptors, chemokines, and growth factors (Figure S4B, Supporting Information). T cells from PBMCs expressed genes associated with T cell development and cytotoxicity, whereas decidual T cells exhibited a gene expression profile related to decidualization and immune regulation (Figure S4C, Supporting Information). B cells in PBMCs predominantly expressed markers associated with activated or naïve B cells (*IGHD*, *IGHM*), while decidual B cells expressed elevated levels of genes related to plasma cell differentiation. Peripheral plasma cells showed higher immunoglobulin A (IgA) expression, whereas decidual plasma cells expressed elevated levels of IgG (Figure S4D, Supporting Information). Additionally, the proportion of plasma cells among all B cells was higher in the decidua (Figure S4E, Supporting Information). Briefly, peripheral immune cells exhibited higher levels of inflammation and activation, whereas decidual immune cells were involved in maintaining homeostasis.

### 2.4 Extensive Immune Dysregulations in PBMCs from OAPS Patients

**Figure 2:**
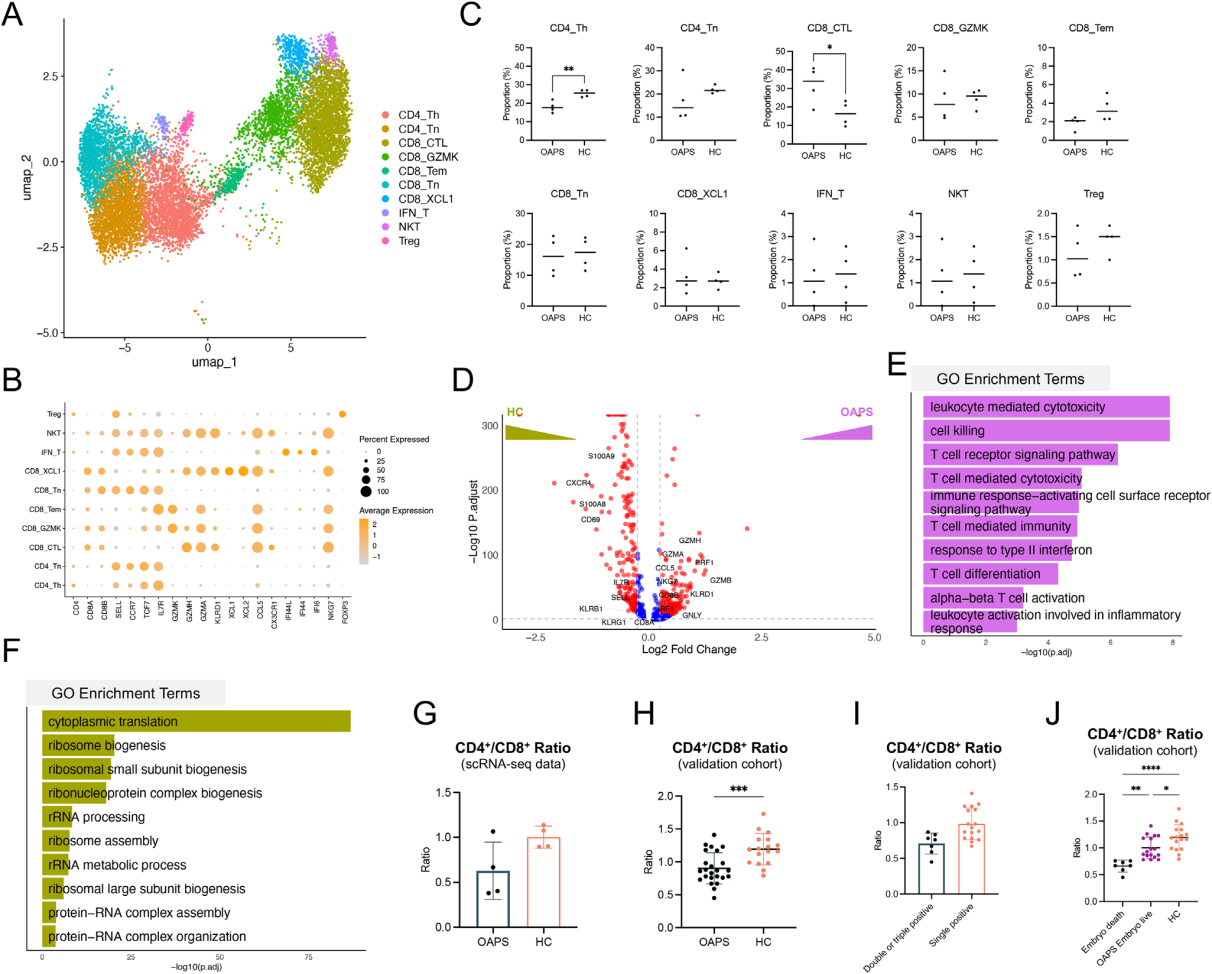
Decreased peripheral CD4^+^/CD8^+^ ratio in OAPS patients compared to HCs. A) UMAP plot depicting the subclusters of T cells in PBMCs. Different colors represent different subclusters. B) Bubble plot of the canonical marker genes among different T subclusters. C) Scatter plots depicting the relative proportions of T subclusters in PBMCs from OAPS patients and those from HCs. D) Volcano plot showing the DEGs of PBMC T cells between OAPS patients and HCs. E) Bar plot showing the enriched GO terms of DEGs in PBMC T cells of OAPS patients. F) Bar plot showing the enriched GO terms of DEGs in PBMC T cells of HCs. G) Bar plot of the CD4^+^/CD8^+^ ratio in PBMCs from OAPS patients and HCs in scRNA‐seq data. H) Bar plot showing the CD4^+^/CD8^+^ ratio in PBMCs from OAPS patients (n = 24) and HCs (n = 17) in the validation cohort. I) Bar plot showing the CD4^+^/CD8^+^ ratio in PBMCs from OAPS patients with single or double/triple positive aPLs. J) Bar plot showing the CD4^+^/CD8^+^ ratio in PBMCs from OAPS patients with embryo demise or embryo live. Data in (C), (G), (H), and (I) are presented as mean ± SD and analyzed using Student's *t*‐test. Data in (J) are presented as mean ± SD and analyzed using one‐way ANOVA with Tukey's multiple comparisons test. ^*^ *p* < 0.05, ^**^ *p* < 0.01, ^***^ *p* < 0.001, ^****^ *p* < 0.0001. DEGs, differentially expressed genes; GO, Gene Ontology; aPLs, antiphospholipid antibodies; OAPS, obstetric antiphospholipid syndrome; HCs, healthy controls; PBMC, peripheral blood mononuclear cells; SD, standard deviation; ANOVA, analysis of variance.

Because the functional changes of decidual immune cells in OAPS have been described,^[^ ^7^ ^]^ we then compared the functional differences of PBMCs immune cells between OAPS patients and HCs. T cells are the most abundant immune cells in PBMCs, and their aberrant homeostasis is associated with pregnancy failure.^[^ ^19^ ^]^ Ten subclusters were identified, namely CD4⁺ naïve T cells (Tn), CD4⁺ helper T cells (Th), CD8⁺ cytotoxic T cells (CTL), CD8⁺GZMK⁺ T cells, CD8⁺ effector memory T cells (Tem), CD8⁺ Tn, CD8⁺XCL1⁺ T cells, IFN⁺ T cells, NKT cells, and regulatory T cells (Treg), based on their marker genes (**Figure** **2**A,B). In OAPS patients, the proportion of CD4⁺ Tn was significantly lower, and the proportion of CD8⁺ CTL was significantly higher than in HCs, while other T cell subclusters showed similar proportions (Figure 2C). By differentially expressed gene (DEG) analysis, distinct expression profiles in PBMCs T cells were found between OAPS patients and HCs (Figure 2D). T cells in OAPS patients expressed elevated levels of cytotoxicity‐related genes, including *NKG7*, *GZMA*, *GZMH*, *PRF1*, and *GNLY*. The upregulated DEGs in OAPS patients were enriched in Gene Ontology (GO) terms such as leukocyte‐mediated cytotoxicity, cell killing, and T‐cell‐mediated immunity (Figure 2E). In contrast, DEGs in HCs were enriched in GO terms related to cytoplasmic translation and ribosome biogenesis (Figure 2F).

Interestingly, based on scRNA‐seq data, the peripheral CD4⁺/CD8⁺ T cell ratio in OAPS patients tended to be lower than that of HCs (Figure 2G). To validate this phenotype, the CD4⁺/CD8⁺ ratio in 24 OAPS patients and 17 HCs from the validation cohort, who had undergone T cell subcluster analysis, was retrospectively analyzed, as detailed in Table S1 (Supporting Information). OAPS patients had a significantly lower CD4⁺/CD8⁺ ratio than HCs (Figure 2H). Patients with double or triple aPL positivity (high‐risk profile) had lower CD4⁺/CD8⁺ ratios (Figure 2I) compared to those with single aPL positivity (low‐risk profile). Peripheral CD4⁺/CD8⁺ ratios were lower in patients with embryonic death than in those with ongoing pregnancies and HCs (Figure 2J), suggesting that a decreased peripheral CD4⁺/CD8⁺ ratio may be a characteristic of OAPS patients and may be associated with disease risk and pregnancy failure.

We then explored the functional differences of PBMCs B cells. Activated B cells, naïve B cells, and plasma cells were identified based on their marker genes (Figure S5A,B, Supporting Information). However, no differences were observed in the proportions of these subclusters (Figure S5C, Supporting Information). B cells in OAPS patients expressed elevated levels of genes related to antigen processing and activation, whereas those in HCs showed greater expression of genes associated with leukocyte cell‐cell adhesion and negative regulation of immune response (Figure S5D–F, Supporting Information). PBMCs myeloid cells can be categorized into cDC1, cDC2, and five types of monocytes based on their marker genes (Figure S6A,B, Supporting Information). In OAPS patients, the proportions of CD16⁺ monocytes and interferon‐response monocytes appeared to be decreased (Figure S6C, Supporting Information). Myeloid cells in OAPS patients exhibited higher expression of genes related to pro‐inflammation, phagocytosis, and apoptosis, while those in HCs exhibited greater expression of genes associated with anti‐inflammation and C‐X‐C like chemokine receptors (Figure S6D, Supporting Information). PBMCs NK cells were divided into CD16⁺ NK, XCL1⁺ NK, and proliferative NK (Figure S7A, Supporting Information). XCL1⁺ NK cells exhibited higher expression of genes related to immune tolerance, such as *KLRC1* and *NCAM1* (Figure S7B, Supporting Information). However, the proportions of these NK cell subtypes between OAPS patients and HCs were similar (Figure S7C, Supporting Information). In summary, extensive disruptions in peripheral immune cells in OAPS patients were found, which may contribute to the pathogenesis of OAPS‐related miscarriage.

### 2.5 Monocyte‐Derived CCR2^+^ Macrophages Infiltrate into OAPS Decidua

**Figure 3:**
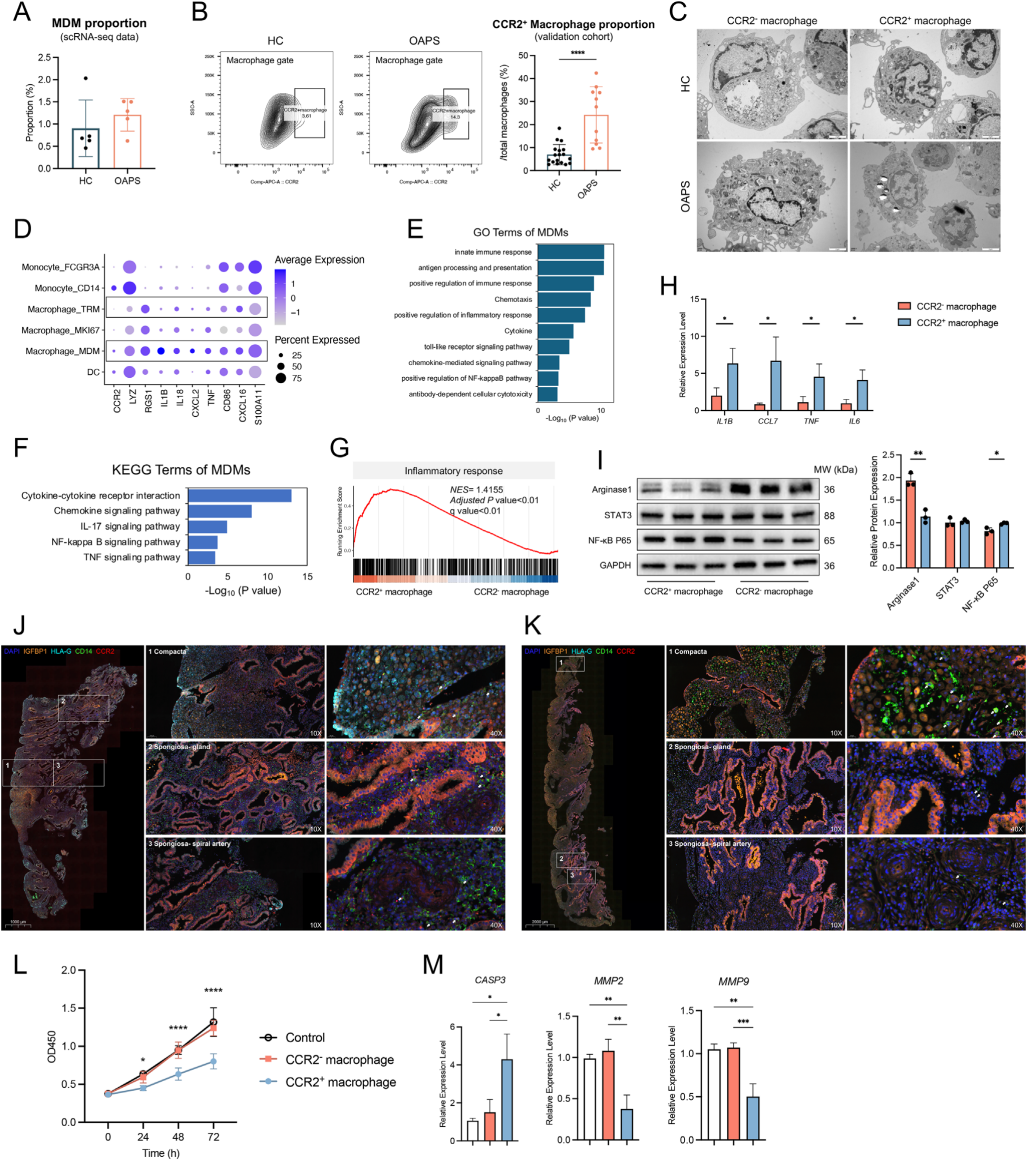
Abnormal infiltration of monocyte‐derived CCR2^+^ macrophages in OAPS decidua and their pro‐inflammatory roles. A) Bar plot showing the proportions of MDMs in decidual immune cells from OAPS patients and HCs in scRNA‐seq data. B) FCM analysis of the proportion of CCR2^+^ macrophages in the decidua from OAPS patients (n = 11) compared with HCs (n = 18). C) Representative images for CCR2^+^ and CCR2^−^ macrophages from the decidua of OAPS patients and HCs under the transmission electron microscope. Scale bar: 2 µm. D) Bubble plot showing the functional gene expressions in different types of decidual myeloid cells based on scRNA‐seq data. E) Bar plot showing the GO terms of marker genes in decidual MDMs. F) Bar plot showing the KEGG terms of marker genes in decidual MDMs. G) GSEA indicates the term of inflammatory response is significantly enriched in decidual CCR2^+^ macrophages. H) Bar plot showing the relative expression levels of genes related to inflammatory factors in decidual CCR2^+^ and CCR2^−^ macrophages, as detected using RT‐qPCR (n = 3). I) Representative immunoblots and semi‐quantified results of Arginase 1, NF‐κB P65, and STAT3 expressions in decidual CCR2^+^ and CCR2^−^ macrophages from OAPS patients (n = 3). J) Representative immunofluorescence images for CD14 (green), CCR2 (red), IGFBP1 (croci), HLA‐G (cyan), and DAPI (blue) co‐staining in decidua from HCs. Scale bar: 1000 µm. K) Representative immunofluorescence images for CD14 (green), CCR2 (red), IGFBP1 (croci), HLA‐G (cyan), and DAPI (blue) co‐staining in decidua from OAPS patients. Scale bar: 2000 µm. L) Line chart showing the effects of medium supernatants from CCR2^+^ and CCR2^−^ macrophages on HTR‐8/SVneo proliferation, as measured by CCK‐8 (n = 3). M) Bar plot showing relative expression levels of *CASP3*, *MMP2*, and *MMP9* genes in HTR‐8/SVneo treated with supernatants from CCR2^+^ and CCR2^−^ macrophages, as detected by RT‐qPCR (n = 3). Data in (A) and (B) are presented as mean ± SD and analyzed using Student's *t*‐test. Data in (H) and (I) are presented as mean ± SD and analyzed using a paired *t*‐test. Data in (L) and (M) are presented as mean ± SD and analyzed using one‐way ANOVA with Dunnett's multiple comparisons test. ^*^ *p* < 0.05, ^**^ *p* < 0.01, ^***^ *p* < 0.001, ^****^ *p* < 0.0001. OAPS, obstetric antiphospholipid syndrome; HCs, healthy controls; MDM, monocyte‐derived macrophage; FCM, flow cytometry; scRNA‐seq, single‐cell RNA sequencing; GO, Gene Ontology; KEGG, Kyoto Encyclopedia of Genes and Genomes; GSEA, Gene Set Enrichment Analysis; RT‐qPCR, real‐time quantitative polymerase chain reaction; SD, standard deviation; ANOVA, analysis of variance.

We further investigated whether the decidual‐infiltrating macrophages in OAPS patients originated from the monocytes that were decreased in PBMCs, this is helpful for us to understand the connections between decidual and peripheral immune cells. As we had anticipated, scRNA‐seq data revealed a higher proportion of MDMs in the decidua of OAPS patients compared with HCs, indicating the flow of monocytes from PBMCs to decidua in OAPS patients (**Figure** **3**A). CCR2 has been confirmed as a surface marker of MDMs in various studies.^[^ ^8^ ^]^ To validate this finding, the proportion of CCR2⁺ macrophages was assessed in a larger validation cohort and was significantly higher in the decidua from OAPS than in that from HCs (Figure 3B).

To further compare the functions of decidual CCR2^+^ MDMs and CCR2^−^ tissue‐resident macrophages (TRMs), CCR2^+^ and CCR2^−^ macrophages were sorted from the decidua of OAPS patients and HCs, then examined using transmission electron microscopy. The gating strategy of FCM is shown in Figure S3B (Supporting Information). CCR2^+^ macrophages were observed to have fewer ruffles, pseudopodia, and organelles (e.g., Golgi, lysosomes) than CCR2^−^ macrophages. These CCR2^−^ macrophages were previously shown to clear tissue debris and regulate the local immune environment.^[^ ^20^ ^]^ Conversely, in the decidua of OAPS patients, CCR2^−^ macrophages were observed to have more ruffles, pseudopodia, and lysosomes than those in HCs, indicating stronger phagocytic activity (Figure 3C). MDMs expressed elevated levels of genes related to inflammatory factors, such as *IL1B*, *IL18*, *CXCL2*, and *TNF*, than TRMs (Figure 3D). The characteristic genes in MDMs were primarily enriched in innate immune response, chemotaxis, antigen presentation, NF‐κB signaling, and TNF‐mediated pathways, which are involved in the production of inflammatory factors and a pro‐inflammatory response (Figure 3E,F). Gene set enrichment analysis (GSEA) revealed that decidual MDMs were more enriched in the inflammatory response compared to TRMs (Figure 3G). However, the characteristic genes in TRMs were mainly enriched in endocytosis, protein transport, lysosome organization, insulin response, and angiogenesis, all of which relate to tissue homeostasis (Figure S8A,B, Supporting Information). We sorted decidual CCR2^+^ and CCR2^−^ macrophages from OAPS patients and validated the expression of *IL1B*, *CCL7*, *TNF*, and *IL6* genes using reverse transcription quantitative polymerase chain reaction (RT‐qPCR), confirming that decidual MDMs expressed higher levels of these genes (Figure 3H).

NF‐κB signaling is crucial for the pro‐inflammatory response of macrophages,^[^ ^21^ ^]^ while arginase 1 is essential for anti‐inflammatory functions.^[^ ^22^ ^]^ NF‐κB P65 expression was found to be significantly higher in CCR2^+^ macrophages, whereas arginase 1 expression was significantly higher in CCR2^−^ macrophages (Figure 3I). The distribution of CCR2^−^ and CCR2^+^ macrophages in the first‐trimester decidua was investigated by multiplex Immunohistochemistry (mIHC). Based on IGFBP1 expression, the decidua compacta (IGFBP1^high^) and decidua spongiosa (IGFBP1^low^) were recognized.^[^ ^23^ ^]^ In HCs, CCR2^+^ macrophages were evenly distributed in the decidua compacta, glandular interstitium, and peri‐spiral arteries. HLA‐G^+^ trophoblasts were observed in the decidua compacta (Figure 3J). In contrast, in OAPS patients, CCR2^+^ macrophages were significantly increased in the decidua compacta but decreased in the glandular interstitium and peri‐spiral arteries. HLA‐G^+^ trophoblasts were rarely detected (Figure 3K).

To investigate the effects of decidual CCR2^+^ macrophages on trophoblast function, primary decidual CCR2^−^ and CCR2^+^ macrophages of OAPS patients were cultured in vitro. The human trophoblast cell line HTR‐8/SVneo was co‐cultured with either the blank medium or conditioned medium from two macrophage subsets. Compared with blank medium and conditioned medium from CCR2^−^ macrophages, conditioned medium from CCR2^+^ macrophages significantly inhibited HTR‐8/SVneo cell proliferation (Figure 3L), upregulated the apoptosis‐related gene *CASP3* expression, and downregulated the expressions of invasion‐related genes *MMP2* and *MMP9* (Figure 3M). CCR2^+^ macrophages may induce pregnancy loss by promoting trophoblast apoptosis and inhibiting proliferation and invasion.

### 2.6 The Ligand of CCR2, CCL2, is Upregulated in the Decidua of OAPS Patients and Mouse Models

**Figure 4:**
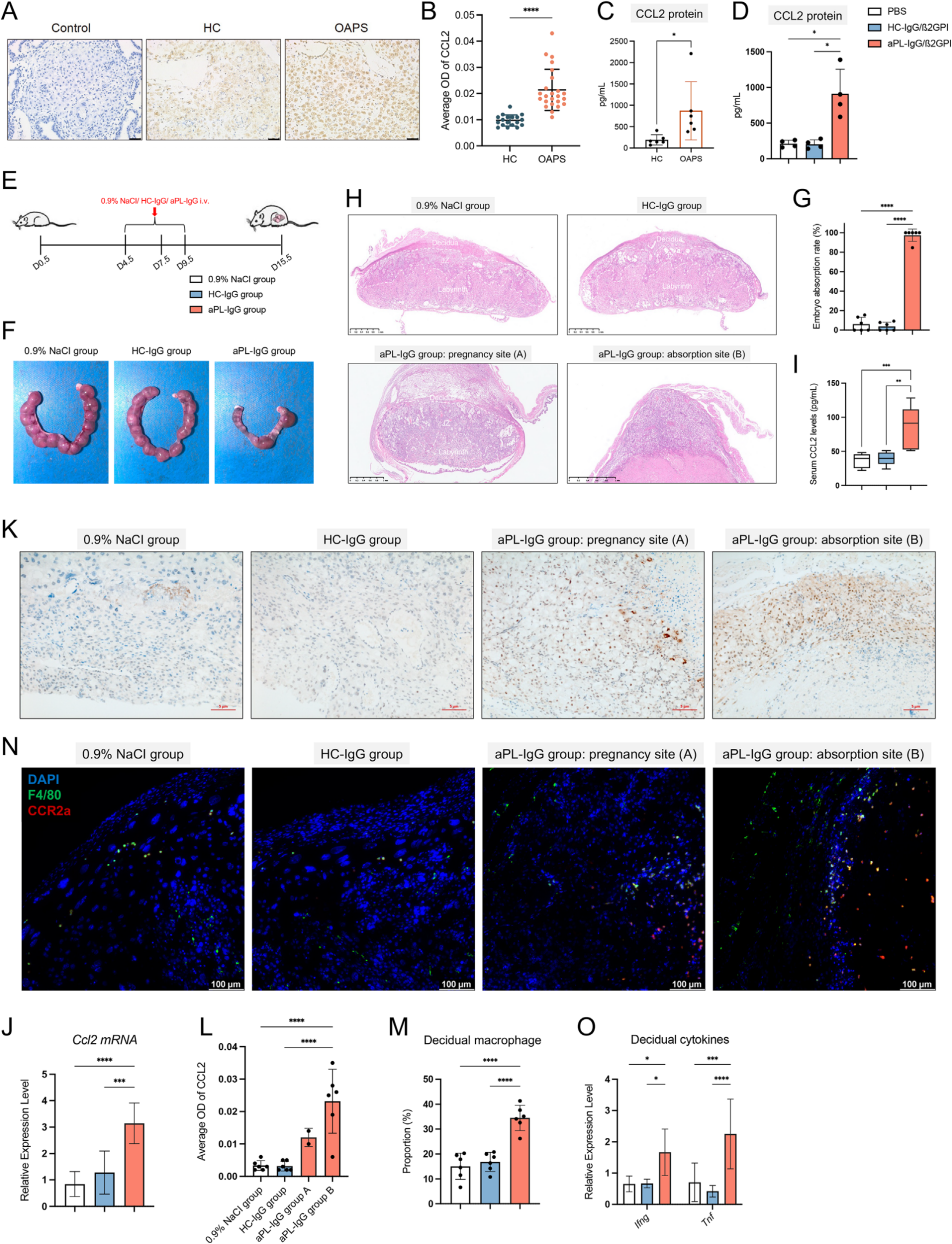
Elevated CCL2 levels in the decidua and serum of OAPS patients and OAPS mice compared with HCs and control mice. A) Representative immunohistochemistry images for CCL2 in decidua from HCs (n = 6) and OAPS patients (n = 8). Three random fields of each sample were used for semiquantitative analyses. Scale bars: 50 µm. B) Scatter plot showing the average OD of CCL2 in decidua. A point represents a random field. C) The expression levels of CCL2 in medium supernatant of primary decidual cells from HCs (n = 6) and OAPS patients (n = 6). D) The expression levels of CCL2 in medium supernatant of primary decidual cells from HCs (n = 4) after stimulation of PBS, HC‐IgG/β2GPI complex, and aPL‐IgG/β2GPI complex. E) Flow chart depicting the methods of establishing OAPS mouse model by intravenous injection of aPL‐IgG. F) Representative figures for the uterus from D15.5 pregnant mice after injection of 0.9% NaCI, HC‐IgG, and aPL‐IgG. G) The embryo absorption rates of D15.5 pregnant mice after injection of 0.9% NaCI, HC‐IgG, and aPL‐IgG (n = 6). H) Representative HE images for decidua and placenta from D15.5 pregnant mice after injection of 0.9% NaCI, HC‐IgG, and aPL‐IgG. The decidua and placenta were detected for sites where embryos were live; for sites where embryos were absorbed, the whole uterus was detected. Scale bars: 1 mm. I) The levels of serum CCL2 in mice of different groups (n = 6). J) The expression of the *Ccl2* gene was detected by RT‐qPCR in the decidua from mice in different groups (n = 6). K) Representative immunohistochemistry images for CCL2 in the decidua from mice of different groups. Three random fields of each sample were used for semiquantitative analyses. Scale bars: 5 µm. L) Bar plot showing the average OD of CCL2 in decidua. A point represents a sample. M) The proportion of macrophages in the decidua of mice from different groups, as detected by FCM (n = 6). N) Representative immunofluorescence images for CCR2^+^ macrophage in decidua from mice of different groups. Scale bars = 100 µm. O) The *Tnf* and *Ifng* gene expression in decidua from mice in different groups, detected by RT‐qPCR (n = 6). Data in (B) and (C) are presented as mean ± SD and analyzed using Student's *t*‐test. Data in (D), (G), (J), (L), (M), and (O) are presented as mean ± SD and analyzed using one‐way ANOVA with Dunnett's multiple comparisons test. For (I), boxplot features: minimum whisker, 25th percentile – 1.5 × IQR or the lowest value within; minimum box, 25th percentile; center, median; maximum box, 75th percentile; maximum whisker, 75th percentile + 1.5 × IQR or the greatest value within. ^*^ *p* < 0.05, ^**^ *p* < 0.001, ^***^ *p* < 0.001, ^****^ *p* < 0.0001. OAPS, obstetric antiphospholipid syndrome; HCs, healthy controls; OD, optical density; aPL, antiphospholipid antibody; Ig, immunoglobin; β2GPI, β2 glycoprotein I; RT‐qPCR, real time‐quantitative reverse transcription polymerase chain reaction; FCM, flow cytometry; IQR, interquartile range; SD, standard deviation; ANOVA, analysis of variance.

We then explored the regulator of decidual MDMs infiltration in OAPS patients. CCL2 is a classic ligand of CCR2, regulating the infiltration of MDMs in various disorders,^[^ ^24^ ^]^ and was found to be overexpressed in the decidua and serum of OAPS compared with HCs.^[^ ^7^ ^]^ Immunohistochemical staining (IHC) revealed that CCL2 expression in the decidua of OAPS patients was significantly higher than in HCs (**Figure** **4**A,B). Primary decidual cells from OAPS patients and HCs were cultured in vitro, and the medium supernatant from OAPS exhibited significantly higher levels of CCL2 (Figure 4C). aPLs and β_2_ glycoprotein I (β2GPI) complex were the pathogenic molecules of OAPS.^[^ ^25^, ^26^ ^]^ Referring to previous studies, IgG was purified from the serum samples of OAPS patients and HCs, simulating aPL‐IgG and HC‐IgG, respectively.^[^ ^27^, ^28^ ^]^ Detailed information on OAPS patients whose serum was used for aPL‐IgG purification is shown in Table S3 (Supporting Information). The primary decidual cells from HCs secreted more CCL2 after the stimulation of aPL‐IgG/β2GPI complex than HC‐IgG/β2GPI (Figure 4D). Considering the absence of trophoblasts in decidual tissue, which are essential components of the maternal‐fetal interface, CCL2 expression in trophoblasts was examined. According to the scRNA‐seq map of placental tissue from the Human Protein Atlas (HPA) database, trophoblasts expressed less of the *CCL2* gene (Figure S9A,B, Supporting Information). The effect of the aPL‐IgG/β2GPI complex on CCL2 expression in trophoblasts was explored using the HTR‐8/SVneo cell line, with results indicating no significant changes in the expression of CCL2 mRNA or protein in trophoblasts following 48 h of stimulation with the aPL‐IgG/β2GPI complex (Figure S9C,D, Supporting Information). Thus, trophoblasts are unlikely to be the primary source of elevated CCL2 in the decidua of OAPS patients.

200 µg aPL‐IgG, 200 µg HC‐IgG, and 0.9% NaCI solution were intravenously injected into pregnant ICR mice at days 4.5, 7.5, and 9.5 of pregnancy to establish OAPS mouse models (Figure 4E). On day 15.5 of pregnancy, there was abundant embryo absorption found in pregnant mice injected with aPL‐IgG, while the mice injected with HC‐IgG or 0.9% NaCI did not demonstrate embryo absorption (Figure 4F,G). In the 0.9% NaCI and HC‐IgG groups, the placenta developed normally. The placenta was insufficient in the aPL‐IgG group, manifesting as abnormal development of the labyrinth layer and expanded junctional zone in the embryo live site, along with no clear placental structure in the embryo absorption site (Figure 4H). Additionally, CCL2 levels were increased in both the serum and decidual tissue of mice in the aPL‐IgG group compared with those in the 0.9% NaCl and HC‐IgG groups (Figure 4I–L). The increased proportions of decidual macrophages and decidual CCR2^+^ macrophages were identified in mice of the aPL‐IgG group with FCM and mIHC (Figure 4M,N; Figure S3C, Supporting Information). Consistent with the findings in clinical samples, *Ifng* and *Tnf* genes, encoding IFN‐γ and TNF‐α respectively, were overexpressed in the decidua of mice from the aPL‐IgG group compared to the other two groups (Figure 4O). These findings demonstrate that established OAPS mouse models can effectively recapitulate the clinical disease phenotype and that CCL2 is upregulated in the decidua of both OAPS patients and mice.

### 2.7 CCL2 Recruits MDMs into the Decidua in OAPS Patients and Mouse Models

**Figure 5:**
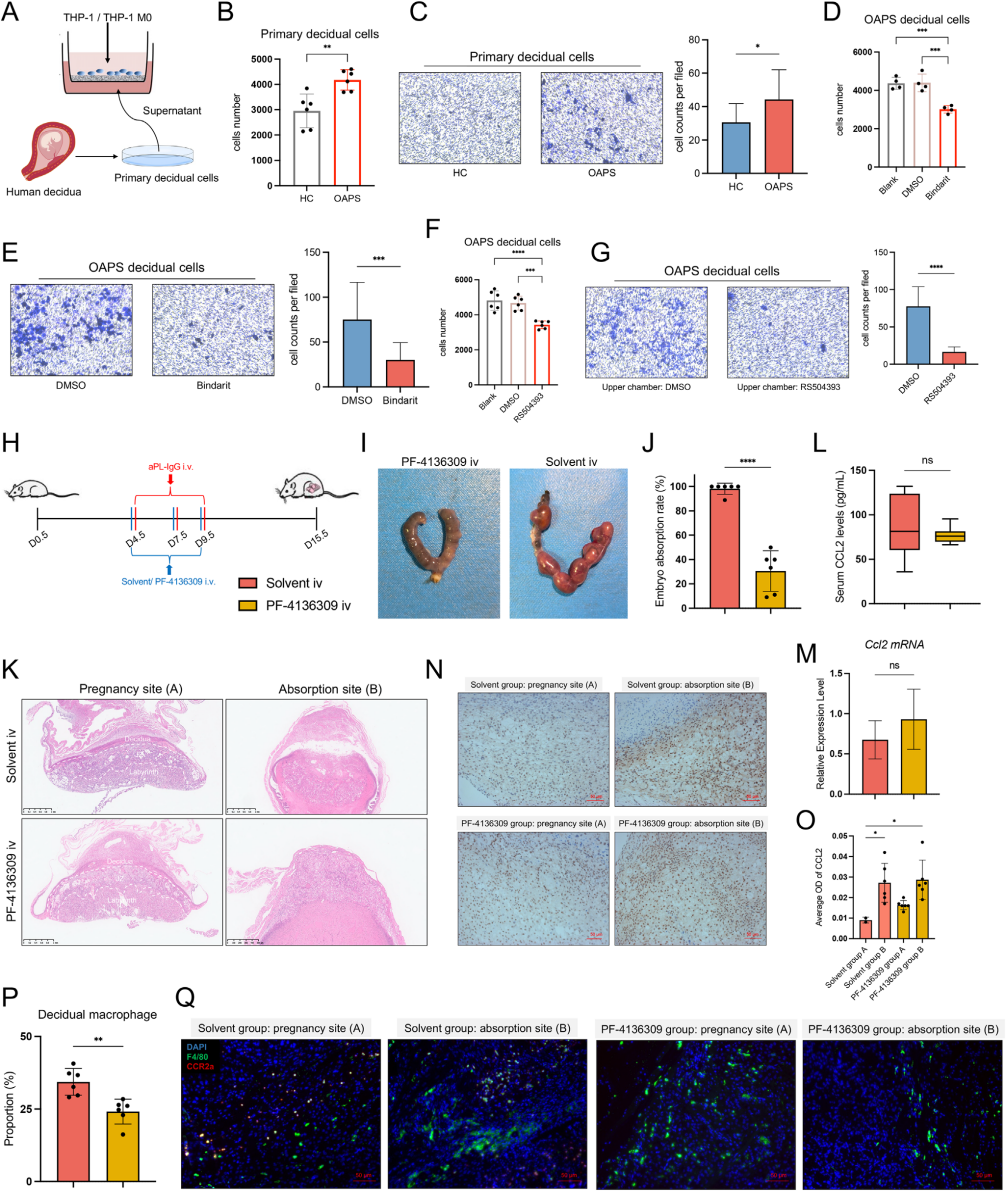
CCL2 promotes CCR2^+^ macrophage infiltration in OAPS decidua. A) Schematic diagram of an in vitro chemotaxis system where primary decidual cells chemoattract THP‐1 or THP‐1‐derived M0 macrophages. Briefly, the culture supernatant of primary decidual cells was added to the lower chamber of a 3.0 µm Transwell insert, and THP‐1 or THP‐1‐derived M0 macrophages, resuspended in FBS‐free medium, were added to the upper chamber. B) Bar plot showing the number of THP‐1 cells in the lower chamber after chemotaxis induced by decidual cells from OAPS patients and HCs (n = 6). C) Representative images and statistical results showing the number of THP‐1 M0 macrophages that migrated through the Transwell membrane (n = 5). Each sample was analyzed in three fields for quantification. D) Bar plot showing the number of THP‐1 cells in the lower chamber after chemotaxis induced by decidual cells from OAPS patients with or without Bindarit treatment (n = 4). E) Representative images and statistical results showing the number of THP‐1 M0 macrophages that migrated through the Transwell membrane (n = 5). Each sample was analyzed in three fields for quantification. F) Bar plot showing the number of THP‐1 cells in the lower chamber after chemotaxis induced by decidual cells from OAPS patients, with or without RS504393 treatment (n = 6). G) Representative images and statistical results showing the number of THP‐1 M0 macrophages that migrated through the Transwell membrane (n = 5). Each sample was analyzed in three fields for quantification. H) Flow chart depicting the methods of treating the OAPS mice model by intravenous injection of PF‐4136309. I) Representative images of uteri from D15.5 pregnant mice after injection of solvent or PF‐4136309. J) Embryo resorption rates in D15.5 pregnant mice following injection of solvent or PF‐4136309 (n = 6). K) Representative HE images for decidua and placenta from D15.5 pregnant mice after injection of solvent and PF‐4136309. L) The levels of serum CCL2 in mice of different groups (n = 6). M) The expression of the *Ccl2* gene was detected by RT‐qPCR in the decidua from mice (n = 5 and 6, respectively). N) Representative immunohistochemistry images for CCL2 in decidua from mice of different groups (n = 6). Three random fields of each sample were used for semiquantitative analyses. Scale bars: 50 µm. O) Bar plot showing the average OD of CCL2 in decidua. A point represents a sample. P) The proportion of macrophage in the decidua of mice that received different treatments was detected by FCM (n = 6). Q) Representative immunofluorescence images for CCR2^+^ macrophage in the decidua from mice that received different treatments. Scale bars: 50 µm. Data in (B), (C), (D), (E), (F), (G), (J), (K), (M), and (P) are presented as mean ± SD and analyzed using Student's *t*‐test. For (K), boxplot features: minimum whisker, 25th percentile – 1.5 × IQR or the lowest value within; minimum box, 25th percentile; center, median; maximum box, 75th percentile; maximum whisker, 75th percentile + 1.5 × IQR or the greatest value within. Data in (O) are presented as mean ± SD and analyzed using one‐way ANOVA with Dunnett's multiple comparisons test. ns: not significant, ^*^ *p* < 0.05, ^**^ *p* < 0.01, ^***^ *p* < 0.001, ^****^ *p* < 0.0001. OAPS, obstetric antiphospholipid syndrome; HCs, healthy controls; RT‐qPCR, real time‐quantitative reverse transcription polymerase chain reaction; ELISA, enzyme‐linked immunosorbent assay; OD, optical density; FCM, flow cytometry; SD, standard deviation; IQR, interquartile range; ANOVA, analysis of variance.

An in vitro model was designed to validate the chemotaxis of monocytes and macrophages from the decidua. Specifically, the medium supernatant of primary decidual cells was added in the lower chamber of a 3‐µm Transwell system, and monocytes or macrophages were cultured in the upper chamber (**Figure** **5**A). THP‐1 is a cell line derived from a patient with acute monocytic leukemia exhibiting a CCR2^‐^positive phenotype.^[^ ^29^, ^30^ ^]^ The medium supernatant from OAPS primary decidual cells exhibited stronger chemotaxis to THP‐1 than that from HCs (Figure 5B). The chemotaxis difference from these medium supernatants to THP‐1‐derived macrophages was also validated (Figure 5C). We repeated the chemotaxis experiments using primary decidual cells from HCs, stimulated with the aPL‐IgG/β2GPI complex, to reach the same conclusion (Figure S10A,B, Supporting Information). To further investigate the role of CCL2, the CCL2 inhibitor Bindarit was added during the culture of primary decidual cells. Bindarit has been proven to significantly inhibit the expression and secretion of CCL2. Decreased chemotaxis was identified from the medium supernatant of both OAPS primary decidual cells and aPL‐IgG/β2GPI complex‐stimulated HC primary decidual cells to THP‐1 and THP‐1‐derived macrophages (Figure 5D,E; Figure S10C,D, Supporting Information). RS504393 is a selective human CCR2 chemokine receptor antagonist and was used to block the CCR2 on the surface of THP‐1, THP‐1‐derived macrophages, and HC primary monocytes in this study. After the pre‐treatment of RS504393, the medium supernatant of OAPS primary decidual cells and aPL‐IgG/β2GPI complex‐stimulated HC primary decidual cells exhibited inhibited chemotactic effects on both THP‐1 and THP‐1‐derived macrophages (Figure 5F,G; Figure S10E,F, Supporting Information). However, no difference was observed in chemotaxis from CCL2 to primary peripheral monocytes between OAPS patients and HCs (Figure S10G,H, Supporting Information).

To explore the effect of CCR2 antagonist PF‐4136309 on OAPS mouse models, PF‐4136309 was intravenously injected 3 h before injecting aPL‐IgG (Figure 5H). PF‐4136309 treatment significantly decreased the embryo absorption rate in OAPS mouse models compared to the solvent group (Figure 5I,J). In the embryo live sites of the PF‐4136309 group, the placenta developed normally (Figure 5K). Interestingly, PF‐4136309 treatment did not significantly change the levels of CCL2 in the serum of OAPS mouse models (Figure 5L). The decidual CCL2 levels in embryo absorption sites were higher in those in embryo live sites, but there was no significant difference between the PF‐4136309 and solvent groups (Figure 5M–O). Notably, PF‐4136309 significantly decreased the proportion of macrophages and CCR2^+^ macrophages in the decidua of OAPS mouse models (Figure 5P,Q). The above results confirmed the regulatory role of CCL2 in decidual CCR2^+^ macrophage infiltration, as well as the potency of CCR2 antagonists in treating OAPS.

### 2.8 Decidual Macrophages are the Main Source of Elevated CCL2

**Figure 6:**
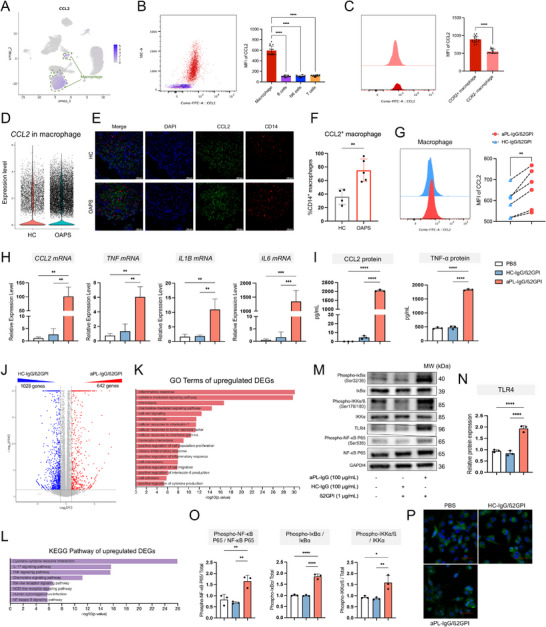
The aPL‐IgG/β2GPI complex promotes macrophages to express more CCL2 via the NF‐κB pathway in vitro. (A) UMAP presenting the expression of *CCL2* gene in the integrated single‐cell landscape. (B) FCM analysis of CCL2 secretion in major decidual immune cells after 18 h LPS stimulation (n = 6). (C) FCM analysis of CCL2 secretion in CCR2^+^ and CCR2^−^ macrophages after 18 h LPS stimulation (n = 6). (D) Violin plot showing CCL2 gene expression levels in decidual macrophages from OAPS patients compared with HCs. (E) Representative immunofluorescence images of CD14 (red), CCL2 (green), and DAPI (blue) co‐staining in the decidua from OAPS patients and HCs. Scale bar: 100 µm. (F) Bar plot showing the proportion of CCL2^+^ macrophages, as determined by semi‐quantitative immunofluorescence staining (n = 4 for HCs, n = 5 for OAPS). (G) FCM analysis of CCL2 secretion in macrophages from the decidua of HCs after stimulation with HC‐IgG/β2GPI and aPL‐IgG/β2GPI complexes (n = 6). (H) Expression levels of *CCL2*, *TNF, IL1B*, and *IL6* genes in THP‐1 M0 macrophages, as detected by RT‐qPCR following stimulation with PBS, HC‐IgG/β2GPI, and aPL‐IgG/β2GPI complexes (n = 3). (I) Levels of CCL2 and TNF‐α in the culture supernatant of THP‐1 M0 macrophages, as detected by ELISA following stimulation with PBS, HC‐IgG/β2GPI, and aPL‐IgG/β2GPI complexes (n = 3). (J) Volcano plot of DEGs in THP‐1 M0 macrophages under the stimulation of HC‐IgG/β2GPI and aPL‐IgG/β2GPI complex. (K) GO terms of upregulated DEGs in (J). (L) KEGG terms of upregulated DEGs in (J). (M) Representative immunoblot showing the expression of TLR4, NF‐κB p65, IκBα, and IKKα in THP‐1 M0 macrophages stimulated with PBS, HC‐IgG/β2GPI, and aPL‐IgG/β2GPI complexes. (N) Bar plot showing the levels of TLR4 in THP‐1 M0 macrophages after different stimulations (n = 3). (O) Bar plot showing the phosphorylation levels of NF‐κB P65, IκBα, and IKKα in THP‐1 M0 macrophages with different stimulations (n = 3). (P) Representative immunofluorescence images for NF‐κB P65 (green) and DAPI (blue) in THP‐1 M0 macrophages after different stimulations. Scale bar: 50 µm. Data in (C) and (G) are presented as mean ± SD and analyzed using a paired *t*‐test. Data in (D) are presented as mean ± SD and analyzed using Student's *t*‐test. Data in (B), (H), (I), (N), and (O) are presented as mean ± SD and analyzed using one‐way ANOVA with Dunnett's multiple comparisons test. ^*^ *p* < 0.05, ^**^ *p* < 0.001, ^***^ *p* < 0.001, ^****^ *p* < 0.0001. OAPS, obstetric antiphospholipid syndrome; HCs, healthy controls; DEGs, differentially expressed genes; aPL, antiphospholipid antibody; Ig, immunoglobin; β2GPI, β2 glycoprotein I; RT‐qPCR, real time‐quantitative reverse transcription polymerase chain reaction; ELISA, enzyme‐linked immunosorbent assay; GO, Gene Ontology; KEGG, Kyoto Encyclopedia of Genes and Genomes; FCM, flow cytometry; ANOVA, analysis of variance; SD, standard deviation.

As CCL2 has been identified as the core regulator of MDMs infiltration, we subsequently investigated the main source of increased CCL2 in the decidua of OAPS patients. The *CCL2* gene is primarily expressed by macrophages (**Figure** **6**A). We designed an FCM protocol to validate the source of CCL2 in decidua (Figure S11A, Supporting Information). Among the main decidua immune cells, macrophages presented the highest mean fluorescence intensity (MFI) of CCL2 (Figure 6B), and CCR2^+^ macrophages had a higher MFI of CCL2 than CCR2^−^ macrophages (Figure 6C). Decidual macrophages from OAPS patients expressed a higher level of the *CCL2* gene (Figure 6D), as validated by mIHC (Figure 6E,F). In addition, the increased MFI of CCL2 after aPL‐IgG/β2GPI complex stimulation was confirmed in decidual macrophages, CCR2^+^ macrophages, and CCR2^−^ macrophages by FCM (Figure 6G; Figure 11B,C, Supporting Information). Then, THP‐1‐derived M0 macrophages were used to explore the effects and mechanisms of aPL‐IgG/β2GPI complex on decidual macrophages. The gene expression levels of *CCL2*, *TNF*, *IL1B*, and *IL6* were significantly higher in macrophages of the aPL‐IgG/β2GPI group than in those of HC‐IgG/β2GPI and blank groups (Figure 6H). Higher levels of CCL2 and TNF‐α in the supernatant from the aPL‐IgG/β2GPI group were detected (Figure 6I). To explore the signaling pathways, mRNA sequencing was performed on treated cells. Significantly different expression profiles were observed between macrophages stimulated with HC‐IgG/β2GPI and aPL‐IgG/β2GPI complexes (Figure S11D, Supporting Information). Compared to the HC‐IgG/β2GPI group, the aPL‐IgG/β2GPI group contained 642 upregulated DEGs (Figure 6J), which were enriched in inflammation, chemotaxis, TNF signaling pathways, Toll‐like receptor‐mediated signaling, and NF‐κB signaling (Figure 6K,L; Figure S11E, Supporting Information). The protein‐protein interaction network showed that *TNF*, *CCL2*, and *NFKBIA* genes were the core upregulated DEGs (Figure S11F, Supporting Information). Graphical representations of the Toll‐like receptor‐mediated pathway showed that TLR4 may contribute to functional changes in macrophages (Figure S11G, Supporting Information). We then detected the expression levels of TLR4 and key molecules in the NF‐κB pathway. TLR4 expression and NF‐κB p65, IKKα, and IκBα phosphorylation levels were significantly higher in the aPL‐IgG/β2GPI group than in other groups (Figure 6M–O). Images taken show NF‐κB p65 entering the nucleus after stimulation with the aPL‐IgG/β2GPI complex (Figure 6P). These findings suggest that the aPL‐IgG/β2GPI complex activates the NF‐κB pathway in macrophages.

**Figure 7:**
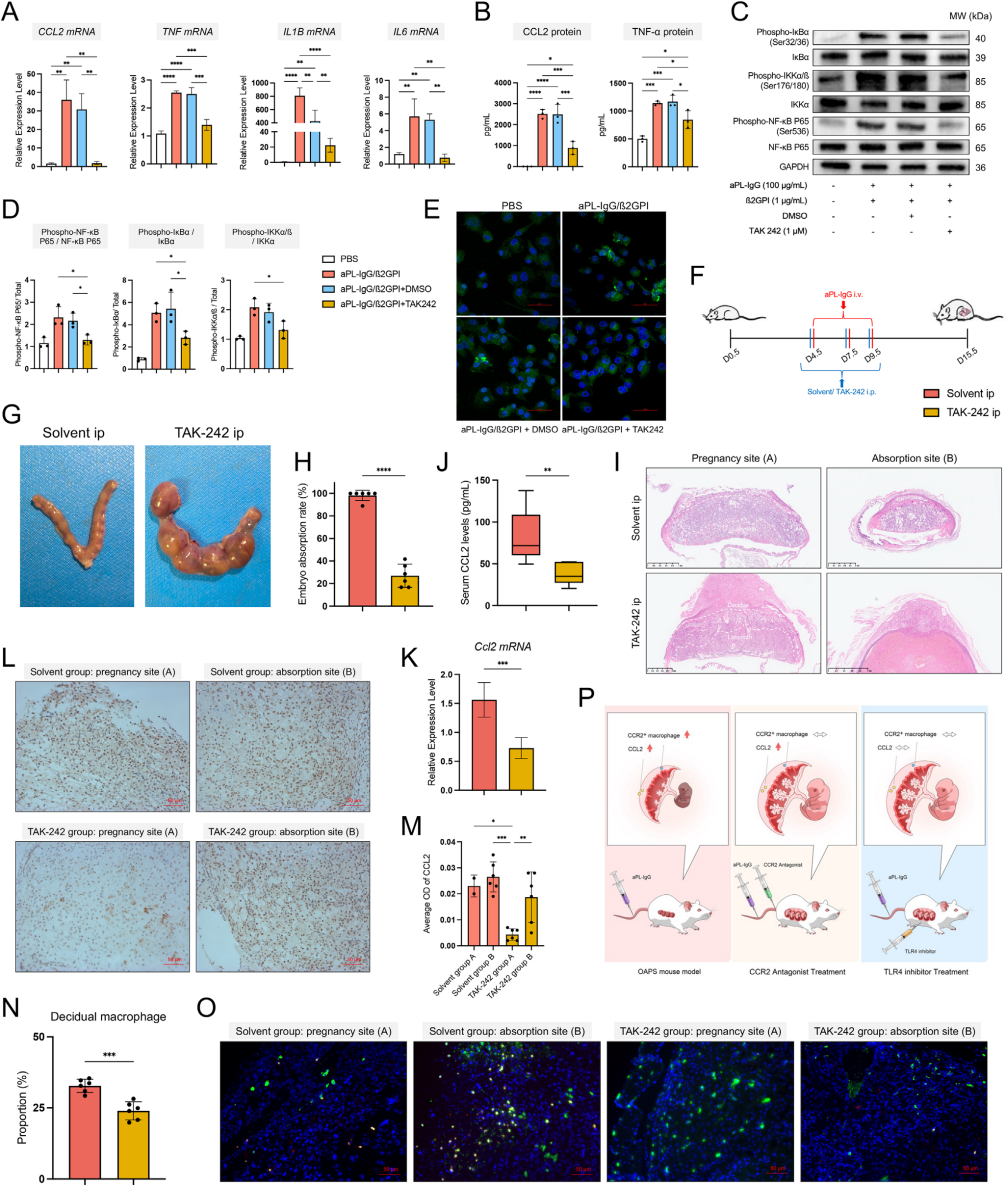
Inhibition of TLR4 decreases CCL2 expression in macrophages in vitro and reduces decidual CCR2^+^ macrophage infiltration and abortion in vivo. (A) Expression levels of *CCL2, TNF, IL1B, and IL6* genes in THP‐1 M0 macrophages stimulated with aPL‐IgG/β2GPI complex and inhibited by TAK‐242, as detected by RT‐qPCR (n = 3). (B) Levels of CCL2 and TNF‐α in THP‐1 M0 macrophage culture supernatant stimulated with aPL‐IgG/β2GPI complex and inhibited by TAK‐242, detected by ELISA (n = 3). (C) Representative immunoblot visualizing the expressions of NF‐κB P65, IκBα, and IKKα in THP‐1 M0 macrophages of different groups (n = 3). (D) Bar plot showing the phosphorylation levels of NF‐κB P65, IκBα, and IKKα in THP‐1 M0 macrophages from different groups (n = 3). (E) Representative immunofluorescence images for NF‐κB P65 (green) and DAPI (blue) in THP‐1 M0 macrophages from different groups. Scale bar: 50 µm. (F) Flow chart depicting the methods of treating the OAPS mice model by intraperitoneal injection of TAK‐242. (G) Representative images of uteri from D15.5 pregnant mice after injection of solvent or TAK‐242. (H) Embryo resorption rates in D15.5 pregnant mice following injection of solvent or TAK‐242 (n = 6). (I) Representative HE‐stained images of decidua and placenta from D15.5 pregnant mice in different groups. (J) The levels of serum CCL2 in mice of different groups (n = 6). (K) Expression levels of *Ccl2* gene in the decidua of mice, as detected by RT‐qPCR (n = 6). (L) Representative immunohistochemistry images showing CCL2 expression in the decidua of mice from different groups. Three random fields per sample were used for semiquantitative analysis. Scale bars: 50 µm. (M) Bar plot showing the average OD of CCL2 in decidua. A point represents a sample. (N) Proportion of macrophages in the decidua of mice with different treatments, as detected by FCM (n = 6). (O) Representative immunofluorescence images showing CCR2^+^ macrophages in the decidua of mice from different groups. Scale bars: 50 µm. (P) Schematic diagram of the OAPS mouse model following treatment with PF‐4136309 and TAK‐242. Data in (H), (I), (K), and (N) are presented as mean ± SD and analyzed using Student's *t*‐test. For (I), boxplot features: minimum whisker, 25th percentile – 1.5 × IQR or the lowest value within; minimum box, 25th percentile; center, median; maximum box, 75th percentile; maximum whisker, 75th percentile + 1.5 × IQR or the greatest value within. Data in (A), (B), (D), and (M) are presented as mean ± SD and analyzed using one‐way ANOVA with Dunnett's multiple comparisons test. ^*^ *p* < 0.05, ^**^ *p* < 0.001, ^***^ *p* < 0.001, ^****^ *p* < 0.0001. OAPS, obstetric antiphospholipid syndrome; HCs, healthy controls; aPL, antiphospholipid antibody; Ig, immunoglobin; β2GPI, β2 glycoprotein I; RT‐qPCR, real time‐quantitative reverse transcription polymerase chain reaction; ELISA, enzyme‐linked immunosorbent assay; TNF, tumor mecrosis factor; FCM, flow cytometry; IQR, interquartile range; SD, standard deviation; ANOVA, analysis of variance.

TLR4 inhibitor TAK‐242 significantly inhibited *CCL2*, *TNF*, *IL1B*, and *IL6* gene expressions induced by the aPL‐IgG/β2GPI complex (**Figure** **7**A). The levels of CCL2 and TNF‐α in culture supernatant were also decreased following TAK‐242 intervention (Figure 7B). Additionally, TAK‐242 significantly decreased the phosphorylation of NF‐κB P65, IKKα, and IκBα (Figure 7C,D). A reduction in NF‐κB p65 in the cell nucleus was also observed, indicating diminished activation of the NF‐κB pathway (Figure 7E). Given the established association between the NF‐κB pathway and the expression of CCL2 and TNF‐α,^[^ ^9^ ^]^ we concluded that the aPL‐IgG/β2GPI complex promotes the CCL2 and TNF‐α expression in decidual macrophages via the TLR4‐NF‐κB pathway.

### 2.9 Inhibiting TLR4 Reduces Decidual CCR2^+^ Macrophage Infiltration and Embryo Absorption

TAK‐242 was used to treat OAPS mouse models. From day 3.5 to day 10.5 of pregnancy, 3 mg kg^−1^ TAK‐242 or solvent was intraperitoneally injected daily (Figure 7F). TAK‐242 significantly decreased the embryo absorption rate of OAPS mouse models (Figure 7G,H). The placenta developed normally in the embryo live sites of OAPS mice treated with TAK‐242. However, in embryo absorption sites, the placenta still developed abnormally (Figure 7I). TAK‐242 decreased the CCL2 level in the serum of OAPS mouse models (Figure 7J). Decidual *CCL2* gene expression was reduced by TAK‐242 treatment (Figure 7K). In embryo live sites of OAPS mice treated with TAK‐242, the level of decidual CCL2 was significantly inhibited; however, in embryo absorption sites, the decidual CCL2 level was similar to that of OAPS mice with solvent intervention (Figure 7L,M). The proportion of decidual macrophages was decreased in OAPS mice after TAK‐242 treatment (Figure 7N). CCR2^+^ macrophages in the decidua of OAPS mouse models with TAK‐242 treatment were reduced (Figure 7O). These findings suggest that the TLR4 inhibitor may reduce decidual CCR2^+^ macrophage infiltration and improve pregnancy outcomes in OAPS mouse models.

### 2.10 Vascular Endothelial Cells Gather CCL2 on the Surface via ACKR1

**Figure 8:**
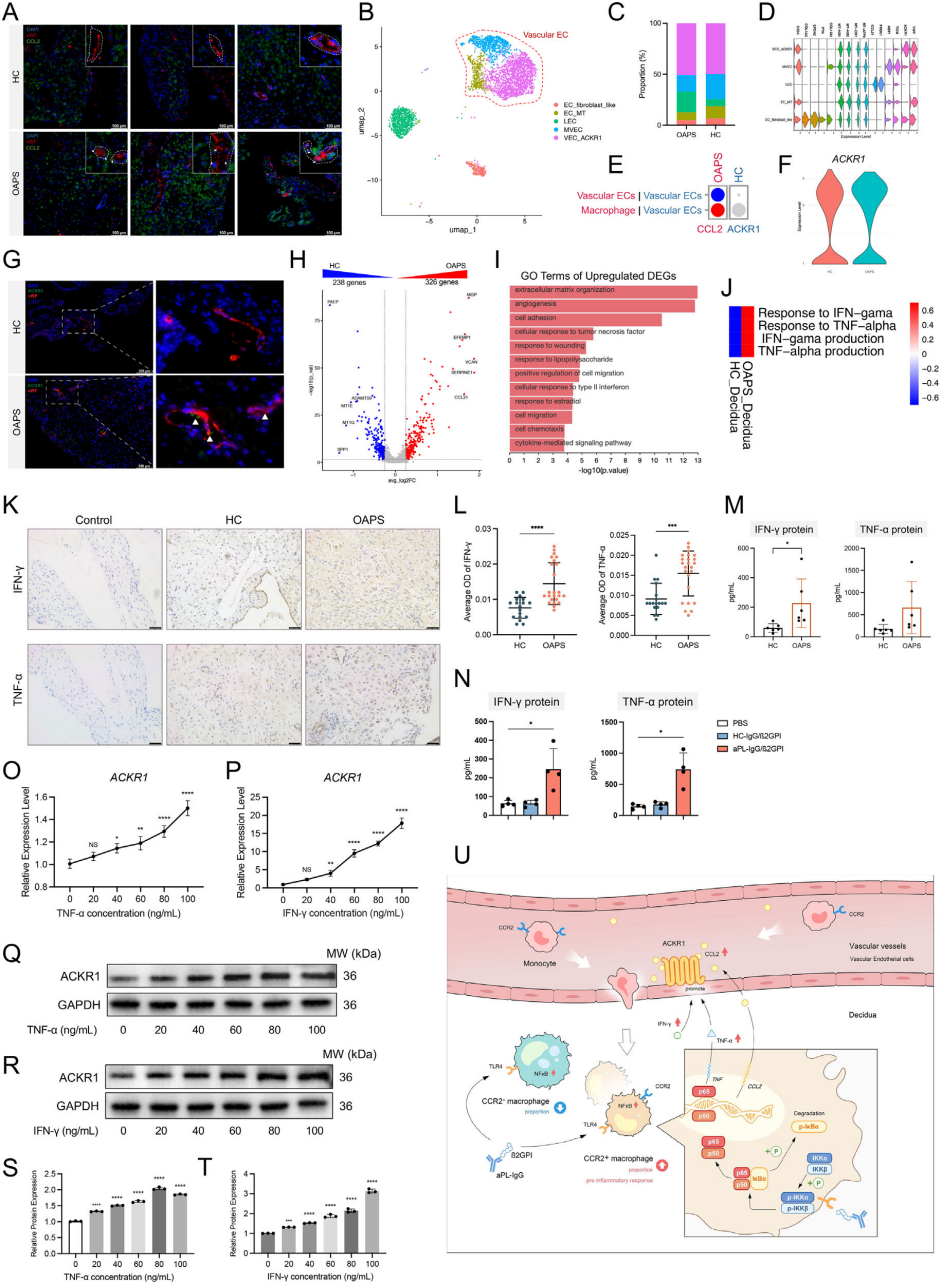
Vascular ECs express higher levels of ACKR1 to aggregate CCL2. (A) Representative immunofluorescence images for vWF (red), CCL2 (green), and DAPI (blue) co‐staining in decidua from OAPS patients and HCs. Scale bar: 100 µm. (B) UMAP plot of EC subclusters at single‐cell resolution. (C) Bar plot showing the proportions of EC subclusters in the decidua of OAPS patients and HCs. (D) Violin plot showing the marker genes of EC subclusters. (E) Bubble plot showing the intercellular communications mediated by ACKR1 and CCL2 between macrophages and vascular ECs in decidua from OAPS patients and HCs. (F) Violin plot of the expression of the ACKR1 gene in vascular ECs of OAPS patients compared with those of HCs decidua. (G) Representative immunofluorescence images for vWF (red), ACKR1 (green), and DAPI (blue) co‐staining in decidua from OAPS patients and HCs. Scale bar: 100 µm. (H) Volcano plot of DEGs for vascular ECs from OAPS patients’ decidua and those from HCs decidua. (I) GO terms of the upregulated DEGs in (H). (J) Heatmap showing the functional scores of vascular ECs in the OAPS and HC groups. (K) Representative immunohistochemistry images for IFN‐γ and TNF‐α in decidua from HCs (n = 6) and OAPS patients (n = 8). Three random fields of each sample were used for semiquantitative analyses. Scale bars: 50 µm. (L) Scatter plot showing the average OD of IFN‐γ and TNF‐α in the decidua. Each point represents a random field. (M) Expression levels of IFN‐γ and TNF‐α in the supernatant of primary decidual cells from HCs (n = 6) and OAPS patients (n = 6). (N) Expression levels of IFN‐γ and TNF‐α in the supernatant of primary decidual cells from HCs following stimulation with PBS, HC‐IgG/β2GPI complex, and aPL‐IgG/β2GPI complex (n = 4). (O) Line chart showing ACKR1 gene expression in EA.hy926 cells under different concentrations of TNF‐α (n = 3). (P) Line chart showing ACKR1 gene expression in EA.hy926 cells under different concentrations of IFN‐γ (n = 3). (Q, S) Representative immunoblot and semi‐quantified results showing ACKR1 protein expression in EA.hy926 cells under different concentrations of TNF‐α (n = 3). (R, T) Representative immunoblot and semi‐quantified results showing ACKR1 protein expression in EA.hy926 cells under different concentrations of IFN‐γ (n = 3). (U) Schematic diagram summarizing the findings of this study. In brief, the aPL‐IgG/β2GPI complex promotes decidual macrophages to express CCL2 via the TLR4‐NF‐κB pathway. CCL2 accumulates on the surface of vascular ECs through ACKR1, recruiting more monocytes into the decidua. Infiltrated CCR2^+^ macrophages exacerbate decidual inflammatory injury in OAPS patients. ACKR1 overexpression in vascular ECs is driven by increased IFN‐γ and TNF‐α levels in the decidual environment of OAPS patients. Data in (L) and (M) are presented as mean ± SD and analyzed using Student's *t*‐test. Data in (N), (O), (P), (S), and (T) are presented as mean ± SD and analyzed using one‐way ANOVA with Dunnett's multiple comparisons test. ^*^ *p* < 0.05, ^**^ *p* < 0.01, ^***^ *p* < 0.001, ^****^ *p* < 0.0001. OAPS, obstetric antiphospholipid syndrome; HCs, healthy controls; aPL, antiphospholipid antibody; Ig, immunoglobin; β2GPI, β2 glycoprotein I; EC, endothelial cell; OD, optical density; TNF, tumor necrosis factor; IFN, interferon; ELISA, enzyme‐linked immunosorbent assay; DEGs, differential expressed genes; SD, standard deviation; ANOVA, analysis of variance.

Increased CCL2 was observed on the surface of decidual vascular endothelial cells (ECs) in OAPS patients but not in HCs (**Figure** **8**A). To explore the underlying mechanisms, the ECs from scRNA‐seq data were re‐analyzed. Decidual ECs were divided into lymphatic ECs (LEC, *PROX1*, *CCL21*), fibroblast‐like ECs (*COL1A2*, *SPON2*), and three types of vascular ECs (*VWF*) (Figure 8B–D). By using the CellPhone DB database, enhanced intercellular communication mediated by macrophage‐derived CCL2 and vascular ECs‐derived ACKR1 was identified (Figure 8E). The *ACKR1* gene was overexpressed in decidual vascular ECs of OAPS patients compared with HCs (Figure 8F). ACKR1 is a key regulator that binds chemokines involved in inflammatory responses.^[^ ^31^, ^32^ ^]^ Its gene expression in decidual vascular ECs was validated by mIHC (Figure 8G). Thus, we hypothesize that decidual vascular ECs may gather CCL2 on the surface via ACKR1 to promote the chemotaxis toward CCR2^+^ monocytes or macrophages.

Previous studies found that aPLs activate vascular ECs in vitro and in vivo^[^ ^33^ ^]^ to promote the expression of adhesive molecules, selectins, and tissue factors.^[^ ^34^, ^35^ ^]^ The effects of the aPL‐IgG/β2GPI complex on ACKR1 expression were validated using the EA.hy926 cell line. However, compared with HC‐IgG/β2GPI control, aPL‐IgG/β2GPI complex did not increase expression levels of ACKR1 and other adhesive molecules (Figure S12A,B, Supporting Information). The mRNA‐seq identified very few DEGs and functional terms between the two groups (Figure S12C–E, Supporting Information). These findings suggest that the aPL‐IgG/β2GPI complex may not be the trigger of ACKR1 expression in vascular ECs.

The gene expression matrix of vascular ECs was extracted from the scRNA‐seq landscape, and the DEGs were analyzed (Figure 8H). The upregulated DEGs of OAPS vascular ECs were enriched in GO in terms of cellular response to TNF and cellular response to type II interferon (Figure 8I). Vascular ECs in OAPS decidua had higher scores of response to IFN‐γ and response to TNF‐α than those in HCs decidua (Figure 8J). We speculate that IFN‐γ and TNF‐α may be associated with ACKR1 expression. By using IHC, increased levels of IFN‐γ and TNF‐α were found in the decidua from OAPS patients compared to those from HCs (Figure 8K,L). The medium supernatant from OAPS primary decidual cells also had significantly higher levels of IFN‐γ and TNF‐α (Figure 8M). The primary decidual cells from HCs secreted more IFN‐γ and TNF‐α after the stimulation of the aPL‐IgG/β2GPI complex than the HC‐IgG/β2GPI complex (Figure 8N). Different concentrations of IFN‐γ and TNF‐α stimulated EA.hy926 cells in vitro, identifying increased expressions of ACKR1, VCAN1, PECAM1, and ICAM1, along with the increased concentrations of IFN‐γ and TNF‐α (Figure 8O–T; Figure S12F,G, Supporting Information). Increased IFN‐γ and TNF‐α may contribute to the chemotaxis of CCR2^+^ macrophages by promoting ACKR1 expression in vascular ECs in OAPS patients.

## 3 Discussion

The immune state in the decidual microenvironment of OAPS patients was revealed in our previous study. Decidual macrophage infiltration is a significant characteristic of OAPS and is worthy of in‐depth exploration.^[^ ^7^ ^]^ According to previous studies, we hypothesize that peripheral immune cells may be linked to decidual immune cells, especially peripheral monocytes, which may serve as the source of decidual‐infiltrating macrophages. However, research on the peripheral immune microenvironment of OAPS patients remains limited, especially the connections between peripheral and decidual immune cells.

In this study, we first described an integrated single‐cell atlas of decidua and PBMCs from OAPS patients and HCs. Significant differences were found in immune cells from the decidua and PBMCs. The decidual immune cells exhibited higher functions in chemotaxis, angiogenesis, growth support, and immune tolerance, whereas peripheral immune cells had higher functions in protein synthesis, inflammatory response, and immune defense. We also revealed peripheral immune disorders in OAPS patients compared with HCs. In detail, there was an abnormal decrease in the proportion of monocytes and the CD4^+^/CD8^+^ ratio, as well as an abnormal increase in the proportion of T cells. These dysregulations may be associated with embryo demise. The increased proportion of macrophages in the decidua and the decreased proportion of monocytes in PBMCs also suggest MDMs infiltration in the decidua of OAPS patients, which was validated in both the clinical cohort and OAPS mouse models. Thus, immune dysfunctions exist in both the decidual and peripheral microenvironment, and the abnormal flow between peripheral and decidual immune cells may also contribute to the pathogenesis of OAPS.

Subsequently, we validated the functions of MDMs in the OAPS decidua. The increased decidual MDMs contribute to pro‐inflammatory effects and trophoblast dysfunction. Increased CCL2 in the decidua is a regulator of CCR2^+^ macrophages. The aPL‐IgG/β2GPI complex can promote decidual macrophages to express more CCL2 and TNF‐α via the TLR4‐NF‐κB pathway, thereby forming a positive feedback loop with CCL2 secretion and CCR2^+^ macrophage infiltration. Increased IFN‐γ and TNF‐α in the decidua of OAPS patients may promote vascular ECs to express more ACKR1, gathering CCL2 to help the chemotaxis to CCR2^+^ macrophage. The experiments on OAPS mouse models demonstrated the potential therapeutic effects of a TLR4 inhibitor and a CCR2 antagonist on decidual macrophage infiltration and embryo absorption (Figure 7P). Therefore, we constructed a comprehensive narrative, establishing the crosstalk between the decreased peripheral monocytes and the increased decidual MDMs in OAPS patients, and delineating the mechanisms by which OAPS drives decidual MDMs infiltration and leads to miscarriage. A schematic diagram of this process is shown in Figure 8U.

β2GPI‐dependent aPLs are recognized as pathologic antibodies in OAPS.^[^ ^26^ ^]^ According to international guidelines, lupus anticoagulant (LA), anticardiolipin antibody (aCL), and anti‐β2GPI are associated with thrombosis and adverse pregnancy outcomes and are included in the laboratory criteria of APS.^[^ ^36^ ^]^ It is generally agreed that reactivity with the LA assay is mainly mediated by antibodies directed against prothrombin and β2GPI, whereas aCL positivity is primarily caused by β2GPI‐dependent aPLs.^[^ ^37^ ^]^ During pregnancy, abundant β2GPI on decidual cells represents an important target for aPLs.^[^ ^38^ ^]^ We purified IgG from OAPS patients with high‐titer positive aCL‐IgG and/or anti‐β2GPI‐IgG to simulate aPL‐IgG and to stimulate primary decidual cells. The aPL‐IgG/β2GPI complex significantly increased the expression of CCL2 and TNF‐α in decidual macrophages via the TLR4‐NF‐κB signaling pathway. The effects of aPLs on these decidual immune cells have rarely been investigated previously. A previous study used IgG isolated from patients with catastrophic APS and healthy controls to stimulate primary monocytes and found significantly enhanced expression of TNF‐α, IL‐6, and VLA4, indicating the pro‐inflammatory roles of aPL on monocytes.^[^ ^39^ ^]^ The anti‐β2GPI IgG was also confirmed to promote the expression of tissue factor and TNF‐α via the TLR4/MyD88 and TLR4/TRIF pathways using the THP‐1 cell line.^[^ ^40^ ^]^ The independent effects of aPL‐IgG/β2GPI on decidual macrophages have expanded our understanding of the pathogenic impact of aPL‐IgG on maternal‐fetal immunity.

CCL2 is the most available chemotactic factor to macrophages.^[^ ^41^ ^]^ After recruitment, decidual macrophages subsequently produce a wide range of inflammatory mediators, including CCL2, and attract additional macrophages, creating a positive feedback loop.^[^ ^42^ ^]^ When the level of CCL2 moves out of the physiological range, many pregnancy‐related diseases will occur.^[^ ^43^ ^]^ Studies found that the decidual tissues of miscarriage patients expressed higher levels of CCL2 than controls.^[^ ^44^, ^45^ ^]^ Some studies have shown that CCL2 levels are higher in the plasma and placenta of patients with pre‐eclampsia^[^ ^46^ ^]^ and preterm labor.^[^ ^47^ ^]^ Our results were consistent with previous studies that both OAPS and other pregnancy complications, such as spontaneous miscarriage, pre‐eclampsia, and preterm labor, tended to exhibit pro‐inflammatory status in the decidual microenvironment. In addition to its chemotactic function, CCL2 acts as an inflammatory cytokine that directly affects immune cells such as macrophages, promoting local inflammatory responses in many diseases.^[^ ^48^ ^]^ Therefore, in the first‐trimester decidua of OAPS patients, CCL2 may also act synergistically with the aPL‐IgG/β2GPI complex to jointly promote the dysfunction of macrophages. However, this hypothesis requires further studies to be confirmed.

This study is the first to uncover the functions of MDMs in the decidua of OAPS patients. In the first‐trimester decidua, TRMs were the primary macrophage type and usually considered to promote immune tolerance and trophoblast invasion by cytokines IL‐10, CCL18, indoleamine 2,3‐dioxygenase (IDO)^[^ ^49^ ^]^ and tissue remodeling‐related molecules insulin‐like growth factor (IGF)‐1 and fibronectin‐1 during pregnancy.^[^ ^50^, ^51^, ^52^, ^53^ ^]^ Contrary to previous understanding, decidual MDMs promote local inflammation and impair trophoblast function. In the first‐trimester decidua of OAPS patients, the proportion of MDMs is significantly increased in response to the chemotaxis of CCL2. The distribution and structure of decidual MDMs were consistent with those of previously reported CCR2^+^ CD11c^high^ macrophages.^[^ ^54^ ^]^ As reported, CCR2^+^ CD11c^high^ macrophages were usually located close to the extra‐villous trophoblast, but CCR2^−^ macrophages were widespread in the decidua. They also found that CCR2^+^ CD11c^high^ macrophages had a higher pro‐inflammatory status than the other two subtypes.

ECs have long been considered the core pathological target of aPL‐IgG, particularly anti‐β2GPI IgG.^[^ ^27^ ^]^ Numerous studies have documented the adverse effects of anti‐β2GPI IgG on ECs, including the upregulation of various pro‐inflammatory chemokines, including CCL2, CCL20, CXCL3, CX3CL1, CXCL5, and CXCL2, as well as pro‐inflammatory cytokines such as IL‐1β and IL‐8.^[^ ^55^, ^56^, ^57^, ^58^ ^]^ The aPL‐IgG/β2GPI complex has been shown to enhance leukocyte‐endothelial cell adhesion through multiple adhesion molecules.^[^ ^56^ ^]^ Additionally, TLR4, annexin A2, apolipoprotein E receptor 2, and LRP6 have been identified as pathological receptors for the aPL‐IgG/β2GPI complex on ECs.^[^ ^55^, ^59^, ^60^ ^]^ However, no significant influence of OAPS patients‐extracted aPL‐IgG on ECs was found in this study, which may be related to the different antibodies used in the studies. Previous studies have typically used aPL‐IgG from thrombotic APS patients, catastrophic APS patients, or recombinant monoclonal aβ2GPI‐IgG. Although the aPLs used for the clinical diagnosis of OAPS and thrombotic APS are the same, an increasing number of studies have shown that OAPS and thrombotic APS are diseases with distinct pathogenic characteristics and mechanisms.^[^ ^38^ ^]^ Data obtained from in vitro studies suggest that IgG antibodies from patients with either pure vascular or obstetric APS can provoke distinct biological effects. For example, IgG from thrombotic APS but not from OAPS patients can promote the phosphorylation of NFκB and p38‐MAPK and upregulate tissue factor activity in monocytes.^[^ ^61^ ^]^ Studies also found that only IgG from OAPS patients who suffered miscarriages, but not thrombotic APS or HCs, inhibited trophoblasts in vitro.^[^ ^62^ ^]^ Researchers suggest that aPLs in OAPS and thrombotic APS may arise from distinct β2GPI‐dependent subpopulations, each with unique biological effects. However, current solid‐phase assays cannot differentiate these subpopulations.^[^ ^38^ ^]^

In this study, we reported a novel role of decidual vascular ECs in the pathogenesis of OAPS. Elevated levels of inflammatory factors IFN‐γ and TNF‐α in the decidua of OAPS patients promote the expression of various adhesion molecules and ACKR1 on the surface of ECs. ACKR1 is known to aggregate CCL2 on the vessel surface, thereby recruiting more monocyte‐derived CCR2^+^ macrophages.^[^ ^32^ ^]^ The overexpression of ACKR1 in ECs has been reported to facilitate leukocyte infiltration in other diseases under the stimulation of inflammatory factors, such as IL‐8.^[^ ^63^ ^]^ However, the detailed mechanism of ACKR1 expression in ECs has not been thoroughly explored. In addition to stimulating inflammatory factors, ACKR1 expression in ECs is associated with neutrophil cells and the subsequent activation of the NF‐κB pathway.^[^ ^64^ ^]^ Epigenetic and transcriptional regulatory mechanisms, such as DNA methylation, may play a role in ACKR1 expression in ECs. However, the specific involvement of these mechanisms requires further investigation.

In addition to macrophage infiltration, CCL2 expression, inflammatory cytokine production, and ACKR1 expression in endothelial cells identified in this study, other factors such as the complement system, neutrophil extracellular traps (NETs), and additional inflammatory factors or chemokines may also contribute to the pathogenesis of OAPS. Studies have identified complement activation at the maternal‐fetal interface of OAPS patients.^[^ ^65^ ^]^ In OAPS patients, complement C5a activation at the maternal‐fetal interface promotes the recruitment of neutrophils expressing tissue factors, leading to a respiratory burst and trophoblast damage.^[^ ^66^, ^67^ ^]^ Additionally, studies have identified substantial NETs deposition in the intervillous space of the placenta in OAPS patients, which can inhibit trophoblast migration, invasion, and in vitro angiogenesis of vascular ECs.^[^ ^68^ ^]^ Additionally, other chemokines, such as CCL3 and CCL4, were also found to be elevated in the OAPS decidua.^[^ ^7^ ^]^ Although these mechanisms were not addressed in this context, they are of significant interest in the pathogenesis of OAPS.

Aspirin plus low‐molecular‐weight heparin (LMWH) is the recommended protocol for treating OAPS patients throughout pregnancy, according to international guidelines.^[^ ^69^, ^70^ ^]^ Aspirin and LMWH exhibit anticoagulant, antithrombosis, and anti‐inflammatory effects. Aspirin inhibits the expression of adhesive molecules in ECs induced by vascular OAPS patient‐extracted aPL‐IgG.^[^ ^71^ ^]^ Aspirin‐triggered‐lipoxins also inhibited the prostaglandin and some pro‐inflammatory cytokines IFN‐γ and IL‐8 synthesis.^[^ ^72^, ^73^ ^]^ LMWH was shown to improve the aPLs‐mediated inhibition of endometrial angiogenesis^[^ ^74^ ^]^ and to affect the functions of trophoblasts.^[^ ^75^ ^]^ Despite receiving standardized treatment, 10–20% of OAPS patients experience fetal death, and 9–10% of OAPS patients experience pre‐eclampsia.^[^ ^76^, ^77^ ^]^ Such cases are clinically termed refractory OAPS. As per ethical guidelines, all participants in our study received aspirin and LMWH treatments. To some degree, the pathogenesis of OAPS identified in this study may explain the occurrence of refractory OAPS. In addition to the classic thrombosis and ECs injury theory, decidual and peripheral immune dysfunction also warrant focus for refractory OAPS patients. Anti‐inflammatory drugs, such as hydroxychloroquine and glucocorticoids, have been proven to improve the prognosis of refractory OAPS.^[^ ^78^, ^79^, ^80^ ^]^ The mechanisms found here may benefit the treatment of refractory OAPS.

While providing novel insights, this study is subject to certain limitations. The enrolled sample size remains small, precluding a deep exploration of the mechanisms underlying peripheral immune disorders in OAPS patients. Furthermore, while focusing on the chemotaxis of the monocyte‐macrophage system in OAPS patients based on the observed decidual MDMs infiltration, the migration of peripheral and decidual NK, T, and B cells was not investigated. Our study did not directly investigate the influence of overexpressed CCL2 on decidual macrophage function in OAPS. Consequently, it remains unclear whether CCL2 exerts synergistic or antagonistic effects on decidual macrophages in the context of the aPL‐IgG/β2GPI complex, nor was its influence on downstream macrophage signaling via other receptors (e.g., CCR4 and CCR1) explored. While animal models demonstrated therapeutic potential, in vivo targeted treatments for CCL2 inhibitors were not performed in this study, primarily due to concerns regarding CCL2's broad physiological functions. More critically, the effects of targeting CCR2 and TLR4 in OAPS require validation in human participants. The biology of pregnancy in human and mouse decidua may differ significantly; consequently, the observed treatment effects may also differ between species. The systemic use of these small‐molecule inhibitors may lead to side effects in other systems throughout the body. Exploring novel drug packaging and delivery methods based on new materials may potentially address this issue in the future.^[^ ^81^, ^82^, ^83^ ^]^ Future clinical trials are warranted to provide further evidence regarding these therapeutic targets.

In conclusion, this study presents a comprehensive integrated single‐cell atlas of both decidua and PBMCs, providing crucial insights into the peripheral and decidual immune states of OAPS patients during the first trimester of gestation. This work highlights that both decidual and peripheral immune disorders are central to OAPS pathogenesis, underscoring their importance for future investigations. We elucidated the source and detailed mechanism of pathogenic decidual macrophage infiltration in OAPS patients. Consequently, CCR2 and TLR4 emerge as promising therapeutic targets for OAPS. The results of this study not only provide a novel understanding of the immune pathogenesis of OAPS but also facilitate future development of targeted treatments. While acknowledging this preclinical promise, the therapeutic potential of these targets necessitates further validation through additional preclinical and rigorous clinical studies.

## 4 Experimental Section

### Sample Processing

Whole blood samples were collected from four OAPS patients diagnosed with embryo demise and four age‐ and gestational week‐matched healthy pregnant women (scRNA‐seq cohort) using BD Vacutainer EDTA Tubes (BD, USA). PBMCs were isolated by gradient centrifugation with Ficoll‐Paque (GE Healthcare, USA). PBMC samples were also collected from OAPS patients (n = 30) and HCs (n = 17) as a validation cohort. Additionally, serum samples were obtained from six OAPS patients with persistent high‐titer positive aCL‐IgG or aβ2GPI‐IgG and six HCs using BD Vacutainer SiO2 Tubes (BD, USA). The serum samples were stored at −80 °C until further use. Decidual samples were collected from 11 OAPS patients with embryo demise and 21 healthy pregnant women retrospectively. Fresh samples were washed three times with sterile PBS and dissociated into single‐cell suspensions using the Tissue Dissociation Kit, Human (Miltenyi Biotec, Germany) and gentleMACS Octo Dissociator (Miltenyi Biotec, Germany). The cell suspensions were filtered through a 70 µm polystyrene filter and used for in vitro culture or flow cytometry (FCM) analysis.

### Participant Inclusion

The diagnosis of OAPS was based on the updated Sapporo criteria,^[^ ^84^ ^]^ and embryo demise was confirmed per international guidelines.^[^ ^85^ ^]^ LA was detected according to the guidelines of the International Society on Thrombosis and Hemostasis.^[^ ^86^ ^]^ Serum aCL IgG/IgM and aβ2GPI IgG/IgM were detected by chemiluminescent immunoassay; 40 IU L^−1^ is the cut‐off value for high‐titer positivity. Control samples were acquired from healthy pregnant women without previous reproductive disorders or pathological pregnancies, and decidual samples were obtained from HCs who received curettage of the uterine cavity. Ethical approval was obtained from the Ethics Committee of West China Second University Hospital, Sichuan University (No. 2024–034). Written informed consent was obtained from all study participants before collecting tissue samples and clinical data.

### scRNA‐seq and Data Processing

PBMCs from samples in the scRNA‐seq cohort were captured by the Singleron Matrix Single Cell Processing System (Singleron, China) according to the manufacturer's protocol to generate single‐cell libraries. The libraries were constructed using the Illumina NovaSeq 6000 platform. Raw sequencing data of the decidua were collected from the previously published scRNA‐seq dataset,^[^ ^7^ ^]^ which was deposited in the OMIX database (No. OMIX004662). Reads from both PBMCs and decidua scRNA‐seq libraries were aligned to the reference genome GRCh38, followed by quantification and quality control using the CeleScope v1.9.0 tool (Singleron, China). The output gene expression matrices were analyzed by the Seurat package (v5.0) in R software (v4.0.4). Quality control was performed for each included scRNA‐seq sample. The criteria for decidual samples were as follows: 500 < number of features < 7000 and percentage of mitochondrial genes < 50%. For PBMC samples, the criteria were: 500 < number of features < 4000, and percentage of mitochondrial genes < 10%. Gene expression matrices were normalized using the “NormalizeData” function, and 3000 highly variation genes were calculated using the “FindVariableFeatures” function. Data scaling was performed using “ScaleData”. Principal component analysis (PCA) was performed using “RunPCA” to reduce the dimensionality of the datasets. To remove batch effects from different samples, the “IntegrateLayers” function was performed using the “HarmonyIntegration” method. The kBET was then calculated to quantitatively evaluate the effectiveness of batch effect correction using the kBET package (version 0.99.6) in R software (version 4.0.4).

### Cell Clustering and Annotation

Cells were clustered using the “RunUMAP,” “FindNeighbors,” and “FindClusters” functions. Cell cluster markers were identified using “FindAllMarkers.” The acknowledged marker genes annotated these cells. The marker genes were visualized using ggplot2 and the Seurat (V5.0) package in R software (v4.0.4).

### DEG Analysis

DEG analysis was performed using the “Wilcox” method in the Seurat package (v5.0), and the Benjamini‐Hochberg method was used to estimate the false discovery rate. DEGs were selected by log2(fold change) > 0.25 and *P* value < 0.05. Volcano plots were graphed with the ggplot2 package. GO, pathway analysis via the Kyoto Encyclopedia of Genes and Genomes (KEGG) database, and GSEA were performed using the clusterProfiler package.

### Gene Set Scoring

The “AddModuleScore” function in the Seurat package was used to calculate the score of cells in the target functions. The gene sets used in cell scoring were downloaded from the MSigDB database.^[^ ^87^ ^]^

### Intercellular Communications Analysis

The intercellular communications mediated by receptor‐ligand pairs were analyzed using the “CellPhoneDB (V5.1.0)” package in R software.^[^ ^88^ ^]^ Cell‐cell interactions with P value <0.05 and average log expression >0.1 were considered significant and were visualized using the “ScBubblePlot” function.

### IgG Purification

Melon Gel IgG Spin Purification Kit (Thermo Fisher, USA) was used to purify the IgG from serum samples according to the manufacturer's protocol. BCA Protein Assay Kit (Beyotime, China) was used to detect the concentration of purified IgG.

### FCM

FCM was performed according to the manufacturer's protocol. To explore the proportions of immune cells in PBMCs, frozen PBMCs were thawed under 37 °C rapidly and stained with APC/Cyanine7 anti‐human CD45 (BioLegend, USA, #304014), Fixable Viability Stain 700 (BD, USA, #564997), Brilliant Violet 650 anti‐human CD14 (BioLegend, USA, #301836), FIFC anti‐human CD16 (BioLegend, USA, #302006), Percep‐Cy5.5 anti‐human CD19 (BioLegend, USA, #302230), Brilliant Violet 510 anti‐human CD56 (BioLegend, USA, #318340) and PE‐Cy7 Anti‐Human CD3 (BD, USA, #557851). To explore the CCR2^+^ macrophage proportion in the decidua, decidual cells were stained with Fixable Viability Stain 510 (BD, USA, #564406), APC/Cyanine7 anti‐human CD45 (BioLegend, USA, #304014), BV421 Anti‐Human CD14 (BD, USA, #563743) and APC anti‐human 192 (CCR2) (BioLegend, USA, #357208). To explore the macrophage proportion in mouse decidua, Fixable Viability Stain 510 (BD, USA, #564406), PE‐Cy7 CD45 (Elabscience, China, #E‐AB‐F1136H), and FITC F4/80 (Elabscience, China, #E‐AB‐F0995C) were used. To investigate the source of CCL2 in the decidua, 100 ng mL^−1^ LPS (Sigma, USA), Brefeldin A (BioLegend, USA), aPL‐IgG (100 µg mL^−1^), HC‐IgG (100 µg mL^−1^), and human β_2_ glycoprotein I protein (MCE, USA) were used for pre‐treatment for 18 h according to different experimental settings. Fixable Viability Stain 700 (BD, USA, #564997), BV421 Anti‐Human CD14 (BD, USA, #563743), PE Anti‐Human CD3 (BD, USA, #557851), APC/Cyanine7 anti‐human CD45 (BioLegend, USA, #304014), Brilliant Violet 510 anti‐human CD56 (BioLegend, USA, #318340) and APC anti‐human 192 (CCR2) (BioLegend, USA, #357208) were stained for surface protein. Fixation Buffer (BioLegend, USA, #420801) and Intracellular Staining Perm Wash Buffer (BioLegend, USA, #421002) were used to fix and permeabilize, and FITC Anti‐Human CCL2 was stained (eBioscience, USA, #11‐7096‐81). Stained cells were detected by BD FACSAria II Fusion (BD, USA) or BD FACSCelesta (BD, USA).

### Cell Sorting

FCM sorted CCR2^+^ and CCR2^−^ macrophages from decidua. The staining strategy was the same as above. Cytek Aurora Spectral Flow Cytometry and SpectroFlo software (Cytek, USA) were used for FCM sorting.

### HE Staining

HE staining was performed in 4% paraformaldehyde‐fixed and paraffin‐embedded tissues, and 4‐µm sections were selected. The sections were deparaffinized and incubated in hematoxylin, 1% hydrochloric alcohol, 1% ammonia, and eosin successively. The images were captured using a Leica DM2500 light microscope (Leica, Germany).

### IHC

After deparaffinization, the antigens were retrieved through hyperbaric heating and incubated with 3% H2O2 for 20 min. Subsequently, a blocking buffer containing 5% fetal bovine serum (FBS, Gibco, USA) was applied for 2 h. Then, sections were incubated overnight at 4 °C with the primary antibodies including the MCP‐1 Rabbit Polyclonal antibody (Proteintech, China, #26161‐1‐AP, 1:500), TNF‐α Rabbit Polyclonal antibody (Proteintech, China, #26405‐1‐AP, 1:500), IFN‐γ Polyclonal antibody (Proteintech, China, #15365‐1‐AP, 1:1000) and Mcp‐1 Rabbit Polyclonal antibody (ABclonal, China, #A7277, 1:1200). Anti‐rabbit IgG, HRP‐linked Antibody (CST, USA, #7074, 1:1000), Anti‐mouse IgG, HRP‐linked Antibody (CST, USA, #7076, 1:1000) and diaminobenzidine were applied to visualize the antigen. To quantify protein expression, three random fields of each section were captured using a Leica DM2500 light microscope (Leica, Germany) and analyzed quantitatively in ImageJ software (version 1.2; W.S. Rasband, Bethesda, MD).

### mIHC

A mIHC Kit (Absin, #abs50013) based on tyramide signal amplification (TSA) technology was used for mIHC according to the manufacturer's protocol. The section preparation process was like that in IHC. CD14 Mouse Monoclonal antibody (Proteintech, China, #60253‐1‐Ig, 1:500), IGFBP1 Rabbit Polyclonal Antibody (Proteintech, China, #13981‐1‐AP, 1:2000), CCR2b‐specific Polyclonal antibody (Proteintech, China, #16154‐1‐AP, 1:800), HLA‐G Mouse Monoclonal antibody (Proteintech, China, #66447‐1‐Ig, 1:800), MCP‐1 Rabbit Polyclonal antibody (Proteintech, China, #26161‐1‐AP, 1:500), vWF Rabbit Polyclonal Antibody (Proteintech, China, #27186‐1‐AP, 1:300), DARC Polyclonal antibody (Proteintech, China, #55185‐1‐AP, 1:200), F4/80 Polyclonal antibody (CST, USA, #70 076, 1:600) and CCR2a‐specific Polyclonal antibody (Proteintech, China, #16153‐1‐AP, 1:800) were used as primary antibodies. Specific secondary antibodies labeled 520, 570, 620, and 700 were utilized. The stained selections were captured using a Zeiss Axio Imager 2 (Carl Zeiss, Germany) or a Leica DM2500 microscope (Leica, Germany).

### Primary Decidual Cell Culture

Decidual cells were cultured in RPMI 1640 medium (Gibco, USA) supplemented with 10% FBS (Gibco, USA) and 1% penicillin‐streptomycin (Gibco, USA) at 37 °C and 5% CO_2_ for 24 h. Primary decidual cells were stimulated with human β_2_‐glycoprotein I (2 µg mL^−1^, MCE, USA), aPL‐IgG (200 µg mL^−1^), HC‐IgG (200 µg mL^−1^), or CCL2 inhibitor Bindarit (30 µM, MCE, USA) in separate experiments. Supernatants were collected by centrifugation for subsequent analyses. Decidual CCR2^−^ and CCR2^+^ macrophages from OAPS patients were sorted by flow cytometry and cultured in RPMI 1640 medium containing 50 ng mL^−1^ M‐CSF (MCE, USA) for 24 h at 37 °C and 5% CO_2_. Supernatants were stored at ‐80 °C for further analysis.

### Isolation of Peripheral Monocytes

PBMCs were acquired as before. CD14^+^ monocytes were purified by using CD14 magnetic beads (Miltenyi Biotec, Germany). Then, these monocytes were cultured in PRMI 1640 (Gibco, USA) with 10% FBS (Gibco, USA) and 1% penicillin‐streptomycin (Gibco, USA) at 37 °C and 5% CO2 and were used for other experiments within 12 h.

### Transmission Electron Microscopy

Sorted decidual macrophages were fixed in 2.5% glutaraldehyde (Solarbio, China) for 2 h at room temperature. Then, the samples were sent to Chengdu Lilai Biotechnology CO., LTD. for ultrathin sectioning. The ultrathin sections were captured under the JEM‐1400 FLASH transmission electron microscope (JEOL, Japan).

### Cell Lines

The THP‐1 cell line, derived from a patient with monocytic leukemia,^[^ ^39^ ^]^ was obtained from the Laboratory of Rheumatology and Immunology, West China Hospital. THP‐1 cells were cultured in RPMI 1640 medium (Gibco, USA) with 10% FBS and 1% penicillin‐streptomycin at 37 °C and 5% CO_2_. M0 macrophages were induced by treating THP‐1 cells with 200 ng mL^−1^ PMA for over 24 h. The HTR‐8/SVneo and EA.hy926 cell lines were purchased from Zhejiang MeisenCTCC, China. HTR‐8/SVneo cells were cultured in RPMI 1640 medium with 10% FBS and 1% penicillin‐streptomycin, while EA.hy926 cells were cultured in DMEM with 10% FBS and 1% penicillin‐streptomycin, both at 37 °C and 5% CO_2_. Both cell lines were authenticated by STR profiling.

### In Vitro Chemotactic Experiments

An in vitro chemotaxis model was designed using a 3‐µm Transwell system (Corning, USA) to investigate the migration of monocytes and macrophages toward decidual cells or recombinant human CCL2 (MCE, USA). Briefly, the supernatant from primary decidual cells or recombinant human CCL2 was placed in the lower chamber, while monocytes or macrophages were cultured in the upper chamber in RPMI 1640 without FBS. For monocytes, 2 × 10⁴ cells in 200 µL RPMI 1640 were added to the upper chamber, and the number of cells in the lower chamber was counted after 6 h. For M0 macrophages, 2 × 10⁴ cells in 200 µL RPMI 1640 were added to the upper chamber after digestion with StemPro Accutase (Thermo Fisher, USA), and the cells were incubated for 12 h. The Transwell membrane was stained with Crystal Violet Staining Solution (Solarbio, China), and the number of migrated cells was quantified using a Leica DM2500 light microscope (Leica, Germany) and ImageJ software (V1.2; W.S. Rasband, Bethesda, MD) by analyzing three microscopic fields per sample. To investigate the effects of CCR2 on chemotaxis, THP‐1 cells or M0 macrophages were pre‐treated with the CCR2 antagonist RS 504 393 (MCE, USA, 500 nM) for 3 h before the experiment.

### Cell Culture and Intervention

HTR‐8/SVneo cells were seeded at 1.0 × 10⁵ cells per well in 12‐well plates and cultured in RPMI 1640 complete medium. After cells adhered overnight, the medium was replaced with RPMI 1640 complete medium containing 50 ng mL^−1^ M‐CSF (MCE, USA), CCR2^−^ macrophage supernatant, or CCR2^+^ macrophage supernatant. Cells were cultured for 48 h at 37 °C with 5% CO2. To detect cell proliferation by CCK8, HTR‐8/SVneo cells were seeded at 5000 cells per well in a 96‐well plate and cultured in RPMI 1640 complete medium overnight. The medium was then replaced with RPMI 1640 complete medium containing 50 ng mL^−1^ M‐CSF (MCE, USA), CCR2^−^ macrophage supernatant, or CCR2^+^ macrophage supernatant. Cell proliferation was assessed at 0, 24, 48, and 72 h by adding 20 µL of CCK‐8 reagent (Beyotime, China) to each well, incubating for 2 h at 37 °C, and measuring absorbance at 450 nm using a microplate reader. To explore the effects of aPL‐IgG/β2GPI complex on HTR‐8/SVneo cells, human β_2_ glycoprotein I protein (MCE, USA, 2 µg mL^−1^), aPL‐IgG (200 µg mL^−1^), and HC‐IgG (200 µg mL^−1^) were added.

The M0 macrophages were polarized in a 6‐well plate as before. 1 mL RPMI 1640 with 5% FBS and 100 ng mL^−1^ PMA was added into a well. Human β_2_ glycoprotein I protein (MCE, USA, 2 µg mL^−1^), aPL‐IgG (200 µg mL^−1^), HC‐IgG (200 µg mL^−1^), TAK‐242 (MCE, USA, 0.1 µM), and DMSO solvent were added into the culture according to different experiment designs for 48 h. EA.hy926 cells were cultured in DMEM with 10% FBS and were stimulated for 48 h. In addition to the above interventions, different concentrations of human TNF‐α (MCE, USA) and human IFN‐γ (MCE, USA) were used to stimulate ACKR1 expression. After stimulations, culture supernatant and cells were collected for the following experiments.

### Immunofluorescence

In brief, cells were cultured on a slide and fixed using Immunostaining Permeabilization Buffer with Triton X‐100 (Beyotime, China). The slide was blocked in a blocking buffer and stained with NF‐κB p65 Rabbit mAb (CST, USA, #8242, 1:500) overnight at 4 °C. Fluor 488 conjugated Goat Anti‐Rabbit IgG (Elabscience, China, #E‐AB‐1055, 1:200) and DAPI were used for secondary staining. The slide was captured under the STELLARIS 5 confocal microscope (Leica, Germany).

### Western Blot

Samples were homogenized in RIPA buffer (Beyotime, China) with protease and phosphatase inhibitors (AbMole, China) and crushed by ultrasound. The protein samples were extracted by centrifugation at 13,000 g for 15 min at 4 °C and quantified using a BCA kit (Beyotime, China). The loading buffer (Solarbio, China) was used to denature the protein. The protein samples were run on a 4–20% SurePAGE gel (GeneScript, China) and transferred to PVDF membranes (BIO‐RAD, USA) by a transfer buffer (Servicebio, China). After incubation in a rapid blocking buffer (Solarbio, China), the membranes were incubated overnight at 4 °C in primary antibodies. The membranes were washed by TBST and incubated with HRP‐linked Antibody (CST, USA, #7074, 1:5000) or Anti‐mouse IgG, HRP‐linked Antibody (CST, USA, #7076, 1:5000) at room temperature for 1 h. The bands were visualized using a supersensitive ECL Kit (OriScience, China) and a Touch Imager (e‐BLOT, China). The semi‐quantified data were calculated using ImageJ software. The primary antibodies used: IκBα Mouse mAb (CST, USA, #4814, 1:1000), Phospho‐IκBα Rabbit mAb (CST, USA, #2859, 1:1000), Phospho‐NF‐κB p65 Rabbit mAb (CST, USA, #3033, 1:1000), NF‐κB p65 Rabbit mAb (CST, USA, #8242, 1:1000), IKKα Mouse mAb (CST, USA, #11930,1:1000), Phospho‐IKKα/β Rabbit mAb (CST, USA, #2697, 1:1000), GAPDH Mouse mAb (Proteintech, China, #60004‐1‐Ig, 1:5000), TLR4 Rabbit Polyclonal Ab (ABclonal, China, #A11226, 1:1000), Arginase‐1 Rabbit Polyclonal Ab (Proteintech, China, #16001‐1‐AP, 1:1000) and STAT3 Rabbit Polyclonal Ab (Proteintech, China, #10253‐2‐AP, 1:1000).

### qPCR

Total RNA from tissue and cell samples was extracted using the EASYaspin Plue RNA Extracting Kit (Aidlab, China), and concentrations were determined with a NanoDrop Micro Spectrophotometer Instrument (Thermo Fisher, USA). The cDNA libraries were acquired by the iscript cDNA Kit (BIO‐RAD, USA). QuantiNova SYBR Green PCR Kit (QIAGEN, Germany) and the CFX96 Touch Real‐Time PCR Detection System (BIO‐RAD, USA) were used for qPCR.

### ELISA

Mouse MCP‐1 ELISA Kit (Elabscience, China), human MCP‐1 ELISA Kit (RUIXIN Biotech, China), human TNF‐α ELISA Kit (Fine Biotech, China), and human IFN‐γ Kit (Fine Biotech, China) were used to detect the levels of cytokines in the serum or culture supernatant according to the manufacturer's protocols.

### mRNA Sequencing and Data Processing

Total RNA samples were prepared by the above protocol. Before cDNA libraries were established, quality detection was performed. Total RNA ≥ 10 µg, concentration ≥ 100 ng µL^−1^, A260/A280 between 2.0 and 2.2 and A260/A230 ≥ 2 were regarded as eligible. VAHTS Universal V6 RNA‐seq Library Prep Kit for Illumina (Vazyme, China) was used for the cDNA libraries establishment. The libraries were quality‐controlled using an Agilent 2200 and sequenced on an Illumina Novaseq 6000 or DNBSEQ‐T7 with a 150 bp paired‐end run. Before read mapping, clean reads were obtained from the raw reads by removing the adaptor sequences and low‐quality reads. The clean reads were then aligned to GRCh38 using Star.^[^ ^89^ ^]^ HTseq was used to obtain gene counts, and the FPKM method was used to determine gene expression.^[^ ^90^ ^]^ The DESeq2 algorithm filtered the differentially expressed genes;^[^ ^91^ ^]^ P‐value and FDR analysis were subjected to the following criteria: 1) Fold Change > 2 or < 0.5; 2) P value < 0.05, FDR < 0.05. Annotations were downloaded from the KEGG database, NCBI (http://www.ncbi.nlm.nih.gov/), UniProt (http://www.uniprot.org/), and the GO database (http://www.geneontology.org/). Fisher's exact test was applied to identify the significant terms (P value < 0.05). Genes were selected from the enriched biological pathway and used Cytoscape for graphical representations of the pathways.^[^ ^92^ ^]^ A protein‐protein interaction network was drawn using Cytoscape. GSEA analyses were performed using FPKM values to identify the most significant pathways following GO or KEGG gene sets. mRNA‐seq data analysis was performed by CytoNavigator SingleCell Analysis Platform (NovelBio Co., Ltd., China).

### Animals and Treatments

Eight‐week‐old female ICR mice were purchased from Beijing Vital River Laboratory Animal Technology Co., Ltd. (China). Mice were housed in the laboratory animal center of West China Second University Hospital, following the guidelines for the care and use of laboratory animals. All experiments were approved by the Animal Ethics Committee of the hospital (No. 2024–040). In brief, female ICR mice were mated with male ICR mice to induce pregnancy (the day when detecting the vaginal plug was regarded as day 0.5 of pregnancy). The pregnant ICR mice were randomly divided into different experimental groups, with each group consisting of 6 mice. To establish abortion mice models, three groups of pregnant ICR mice were intravenously injected with 200 µg aPL‐IgG (model group), 200 µg HC‐IgG (control group), and 0.9% NaCI solution (blank group) at day 4.5, 7.5 and 9.5 of pregnancy, respectively. To explore the therapeutic effects of CCR2 antagonists on OAPS, the abortion mice models were divided into two groups, 2 mg kg^−1^ PF‐4136309 (MCE, USA) and solvent composed of 10% DMSO, 40% PEG300, 5% Tween‐80, and 45% 0.9% NaCI solution was intravenously injected at 3 h before injection of aPL‐IgG, retrospectively. To explore the therapeutic effects of TLR4 inhibitors on OAPS, the abortion mice models were divided into two groups. Intraperitoneal injection of 3 mg kg^−1^ TAK‐242 and solvent was performed daily from day 4.5 to day 9.5 of pregnancy, retrospectively.

### Mouse Serum and Decidua Collection

Orbital venous plexus blood collection was performed for mice under anesthesia at day 15.5 of pregnancy. Serum samples were acquired by centrifugation of 3000 rpm for 15 min. All mice were sacrificed at day 15.5 of pregnancy to examine and calculate the embryo resorption rate. At the fetal live site, the fetus was removed carefully, and the placenta was fixed in 4% paraformaldehyde. At the embryo absorption site, the whole uterus segment was fixed. Decidual tissues were collected for other experiments, as previously reported.^[^ ^93^ ^]^ Briefly, the decidua was detached from the placenta under a dissecting microscope. The decidual tissues were either frozen or digested with 1 µg mL^−1^ collagenase A (Sigma, USA), 1 µg mL^−1^ collagenase D (Sigma, USA), and 100 ng mL^−1^ DNAse I (Sigma, USA) at 37 °C for 45 min, the cell suspensions were filtered through a 70 µm strainer and used for FCM.

### Statistical Analysis

All statistical analyses were performed using R (v4.2.1) and Prism (v.9). For continuous variables such as gene expression levels, comparisons between two groups were made using the Student's *t*‐test or paired *t*‐test if data were normally distributed, or the Mann‐Whitney U test if data were abnormally distributed. For comparisons among multiple groups, one‐way ANOVA was used for normally distributed data, followed by post hoc tests: Dunnett's test for comparisons of multiple experimental groups with a single control group and Tukey's test for comparisons among multiple groups. The Kruskal‐Wallis H test was used for non‐normally distributed data, followed by Dunn's test for pairwise comparisons. FlowJo (version 10.7) software was used to analyze flow cytometry results. Immunohistochemistry, immunofluorescence images, and Western blot quantification were analyzed using Fiji software. Specific statistical methods were indicated in the figure legends. All experiments were performed with at least three biological replicates from different biological individuals. For experiments using cell lines, at least three independent experiments were conducted at different times to ensure reproducibility. All statistical analyses were two‐tailed, with p<0.05 considered statistically significant.

## Conflict of Interest

The authors declare no conflict of interest.

## Ethical Statement

Ethical approvals were obtained from the ethics committee of West China Second University Hospital, Sichuan University (No. 2024–034). Written informed consent was obtained from all study participants before collecting tissue samples and clinical data. All animal experiments were approved by the Animal Ethics Committee of the hospital (No. 2024–040).

## Author Contributions

R.G. and P.Q. contributed equally to this work. R.G., P.Q., J.H., Z.H., Q.Y., H.L., H.C., Y.T., and R.Z. performed experiments and prepared figures. X.L. and F.Y. collected clinical samples. X.Z. collected clinical information. R.G., C.L., X.Z., and Q.L. designed experiments. R.G. and P.Q. analyzed data. R.G. wrote the manuscript.

## Supporting information



Supporting Information

## Data Availability

The data that support the findings of this study are openly available in [the OMIX, China National Center for Bioinformation / Beijing Institute of Genomics, Chinese Academy of Sciences] at [https://ngdc.cncb.ac.cn/omix], reference number [OMIX008641, OMIX008642 and OMIX008643].
