# Supplementary material for: Deciphering the Role and Mechanism of Decidual Monocyte‐Derived Macrophage Infiltration in Obstetric Antiphospholipid Syndrome at Single‐Cell Resolution
Source: Adv Sci (Weinh). 2025 Aug 7;12(41):e03480. doi: 10.1002/advs.202503480 (PMC12591211; doi:10.1002/advs.202503480)
Supplement: Supplementary file 1 — Supporting Information [file ADVS-12-e03480-s001.pdf]

Supporting Information

Deciphering the Role and Mechanism of Decidual Monocyte-Derived Macrophage Infiltration in Obstetric Antiphospholipid Syndrome at Single-Cell Resolution

Rui Gao[#], Pingying Qing[#], Hanxiao Chen, Zhengyan Hu, Qiaoran Yang, Chenyang Lu, Huimin Liu, Rujun Zeng, Yuanting Tang, Fan Yu, Jinbiao Han[*], Xin Liao[*], Xun Zeng[*], Lang Qin[*]

Supplementary Figures

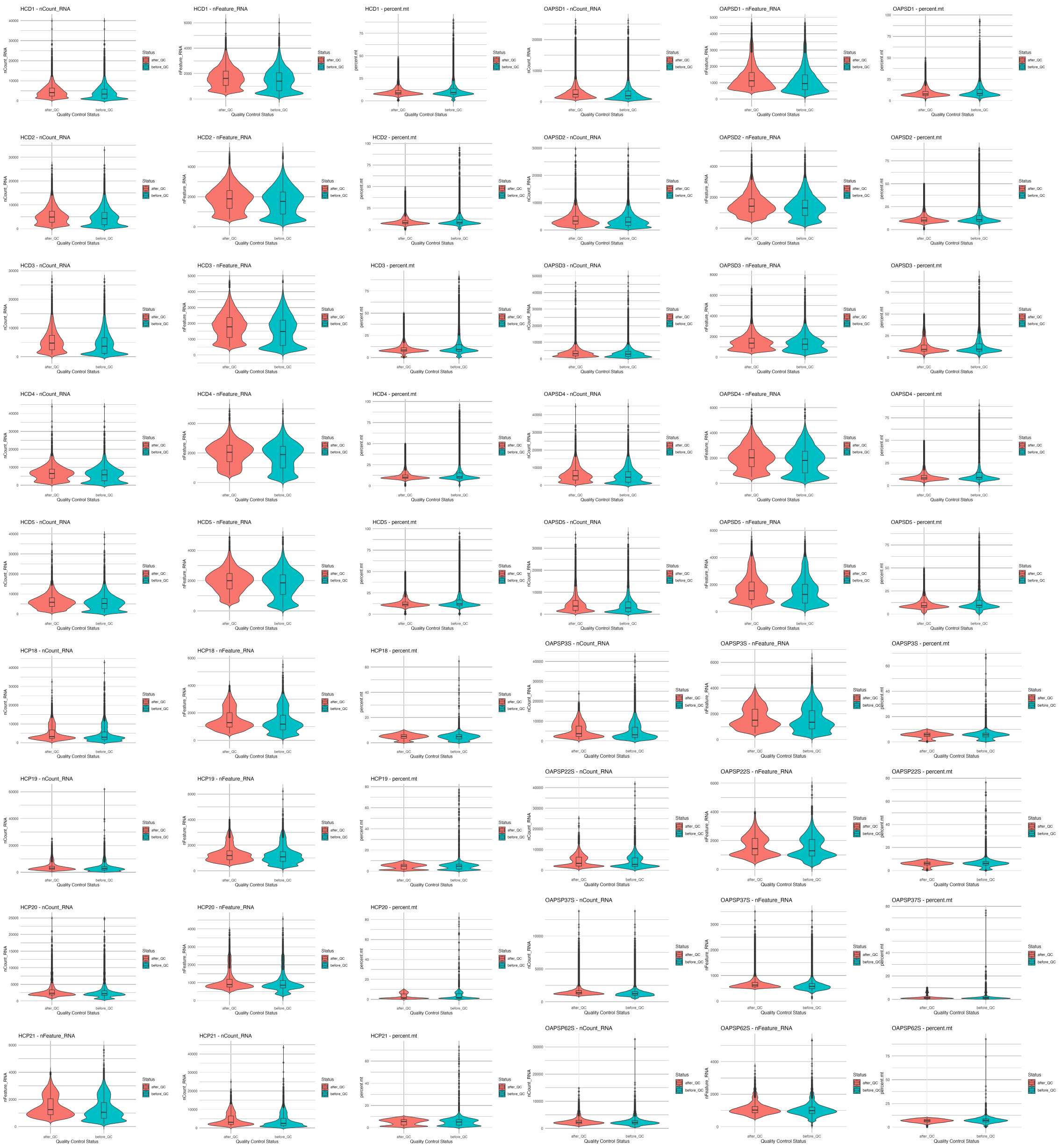

Supplementary Figure 1. Violin plots depicting the number of counts, number of features and percentage of mitochondrial genes before and after quality control for each sample.

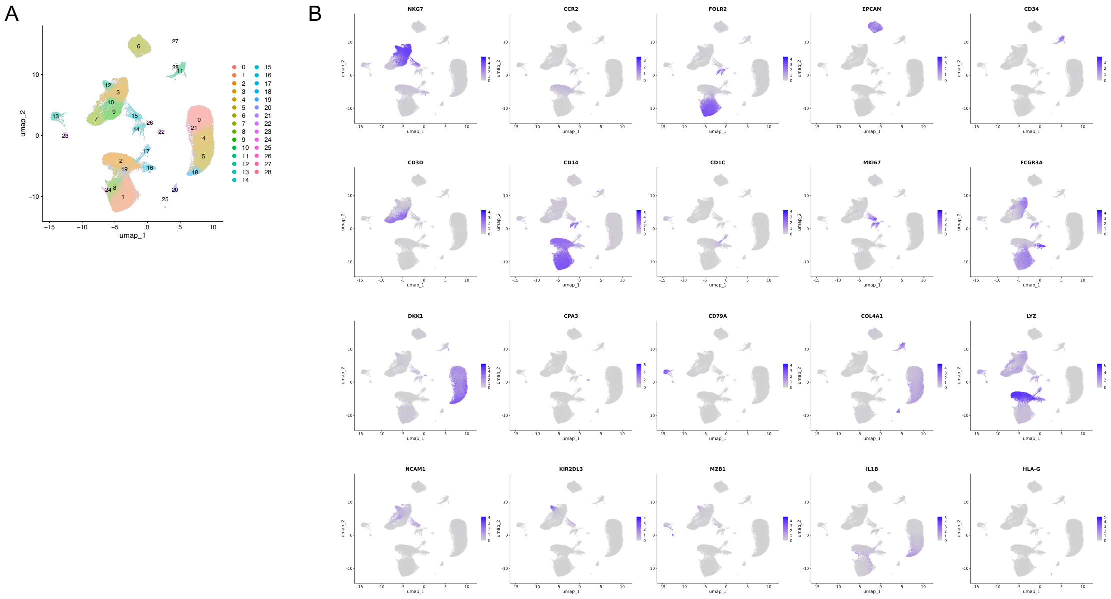

C

B	DC	Endothelial_cell	Epithelial_cell	Fibroblast	Macropahge			Mast_cell	Monocyte		NK				Plasma	Proliferative_cell	Stromal_cell	T	Trophoblast
					Macrophage_MDM	Macrophage_MKI67	Macrophage_TRM		Monocyte_CD14	Monocyte_FCGR3A	NK	NK_KIR2DL3	NK_MKI67	NK_NCAM1					
3202	2137	3806	9415	1306	1342	2836	30262	669	17741	2238	17119	3271	2367	3774	526	166	56483	14361	409

Supplementary Figure 2. Results of clustering, cell type identification and cell proportion analysis.

(A) UMAP plot showing the primary clusters of the integrated single-cell landscape under a resolution of 0.6. (B) UMAP plots showing the expression features of marker genes in the integrated single-cell landscape. (C) Number of cells for each cell type.

OAPS, obstetric antiphospholipid syndrome; HCs, healthy controls; natural killer cell, NK; DC, dendritic cell.

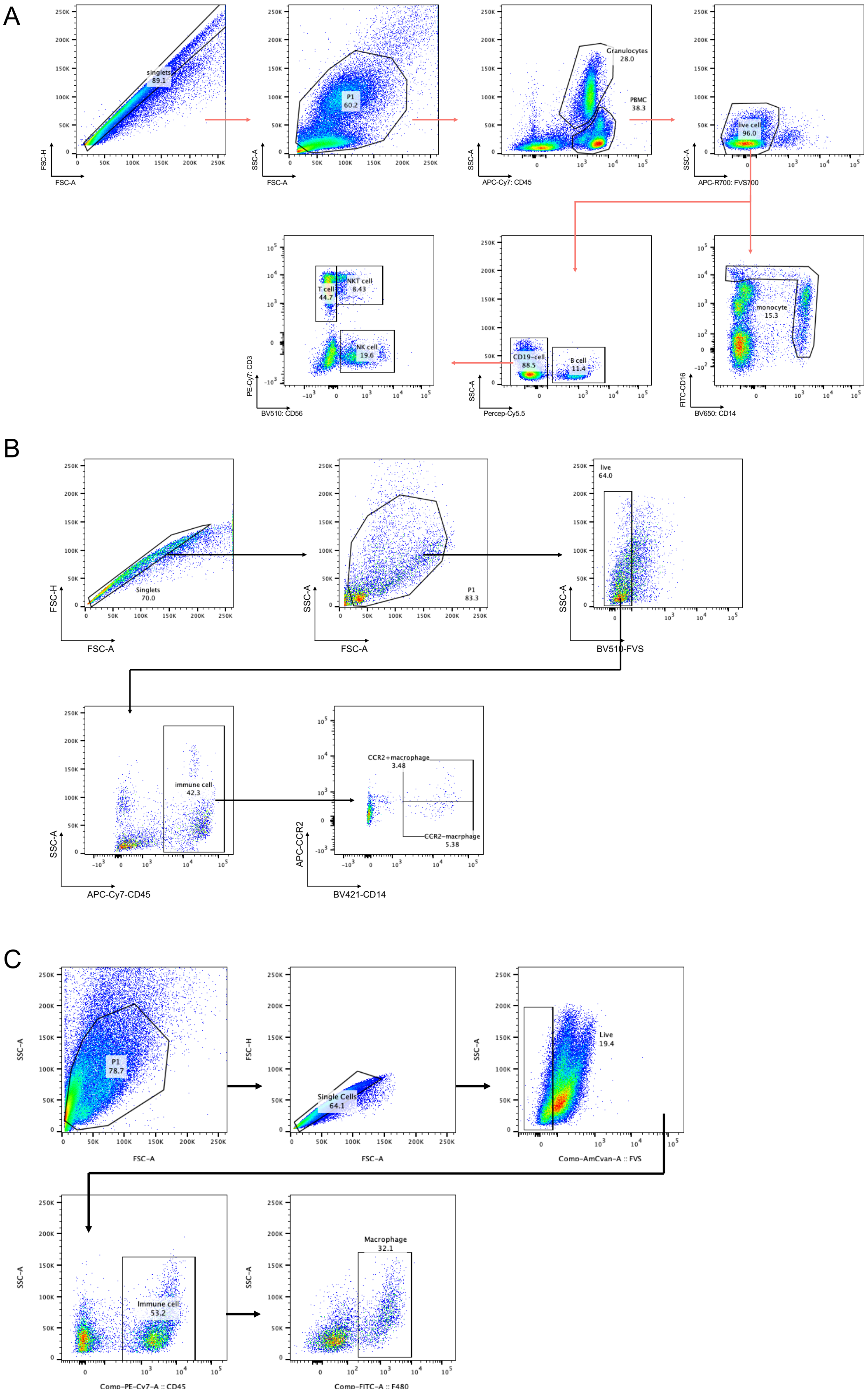

Supplementary Figure 3. Gating strategy for flow cytometry.

(A) Gating strategy for FCM detecting immune cells in human PBMCs. (B) Gating strategy for FCM detecting and sorting CCR2^{+} and CCR2^{-} macrophages in human decidual samples. (C) Gating strategy for FCM detecting macrophages in mouse decidua.

FCM, flow cytometry; PBMC, peripheral blood mononuclear cell.

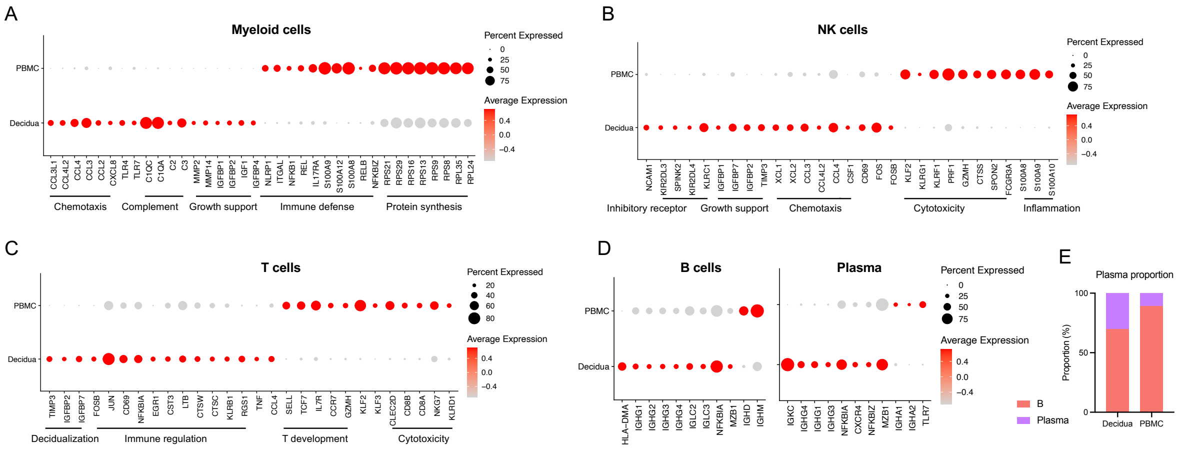

Supplementary Figure 4. Functional differences of the immune cells between PBMCs and decidua.

(A) Bubble plot depicting the functional gene expressions of myeloid cells in decidua and PBMCs. (B) Bubble plot illustrating the functional gene expressions of NK cells in decidua and PBMCs. (C) Bubble plot showing the functional gene expressions of T cells in decidua and PBMCs. (D) Bubble plot showing the functional gene expressions of B and plasma cells in decidua and PBMCs. (E) Bar plot comparing the proportions of B cells and plasma cells in decidua and PBMCs.

PBMC, peripheral blood mononuclear cells; NK, natural killer cells.

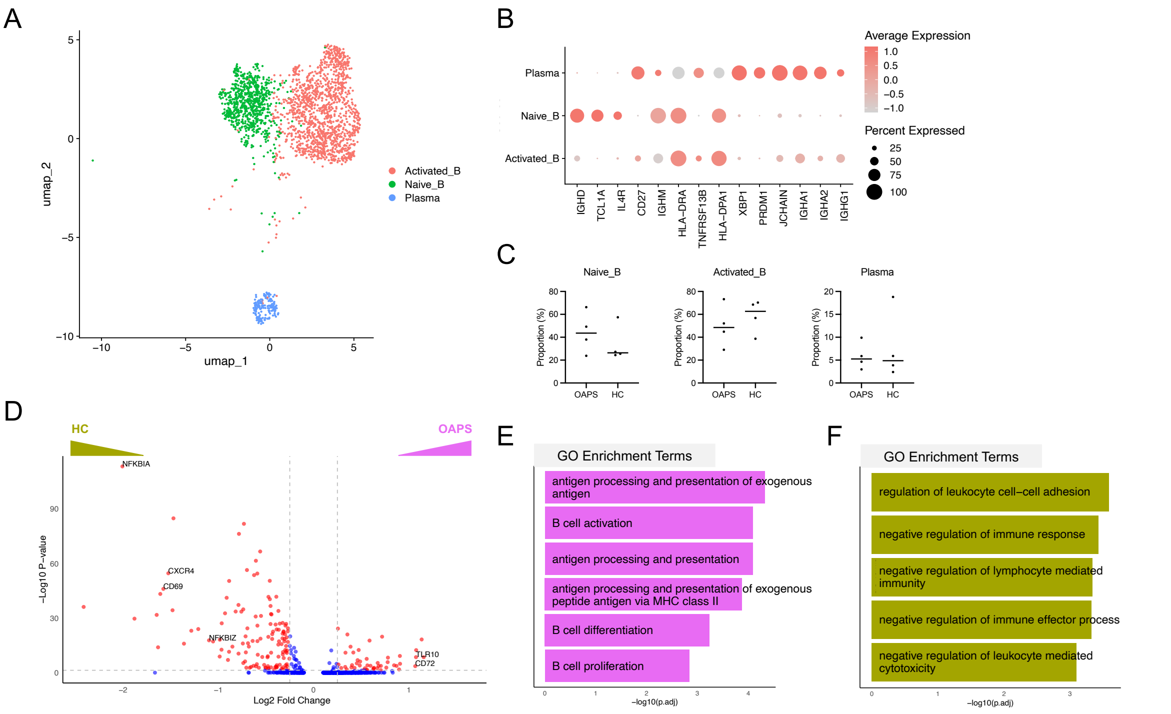

Supplementary Figure 5. The functional characteristics of B cells in PBMCs from OAPS patients and HCs.

(A) UMAP plot of B subclusters in PBMCs. Different colors represent different subclusters. (B) Bubble plot showing the expressions of canonical marker genes. (C) Scatter plots showing the proportions of B cell subclusters among total B cells in PBMCs from OAPS patients and HCs. (D) Volcano plot depicting the DEGs of total B cells in PBMCs between OAPS patients and HCs. (E) Bar plot showing the enriched GO terms of DEGs in OAPS patients. (F) Bar plot showing the enriched GO terms of DEGs in HCs.

OAPS, obstetric antiphospholipid syndrome; HCs, healthy controls; PBMCs, peripheral blood mononuclear cells; DEGs, differentially expressed genes

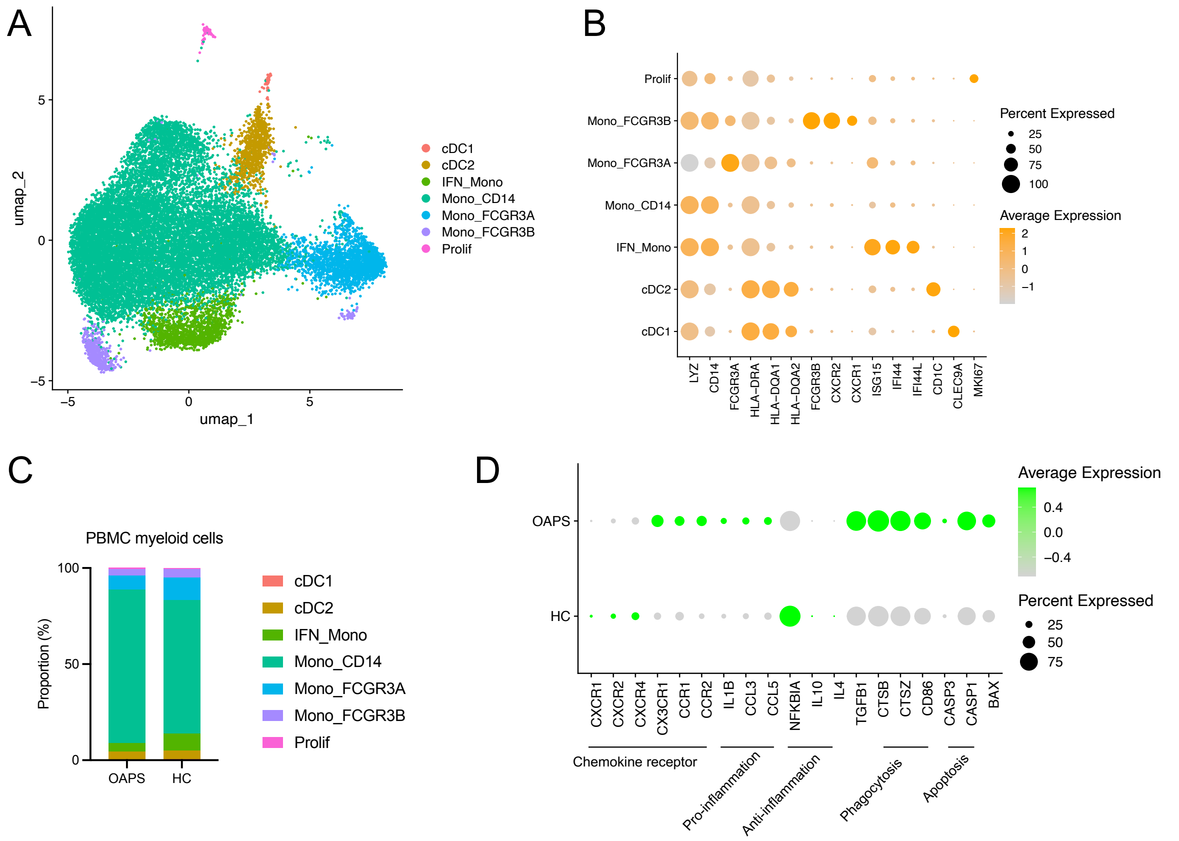

Supplementary Figure 6. The functional characteristics of myeloid cells in PBMCs from OAPS patients and HCs.

(A) UMAP of myeloid subclusters in PBMCs. Different colors mean different subclusters. (B) Bubble plots showing the expressions of canonical marker genes. (C) Bar plot of the proportions of myeloid subclusters in PBMCs from OAPS patients and those from HCs. (D) Bubble plot showing the functional genes of myeloid cells in PBMCs from OAPS patients and HCs.

OAPS, obstetric antiphospholipid syndrome; HCs, healthy controls; PBMCs, peripheral blood mononuclear cells.

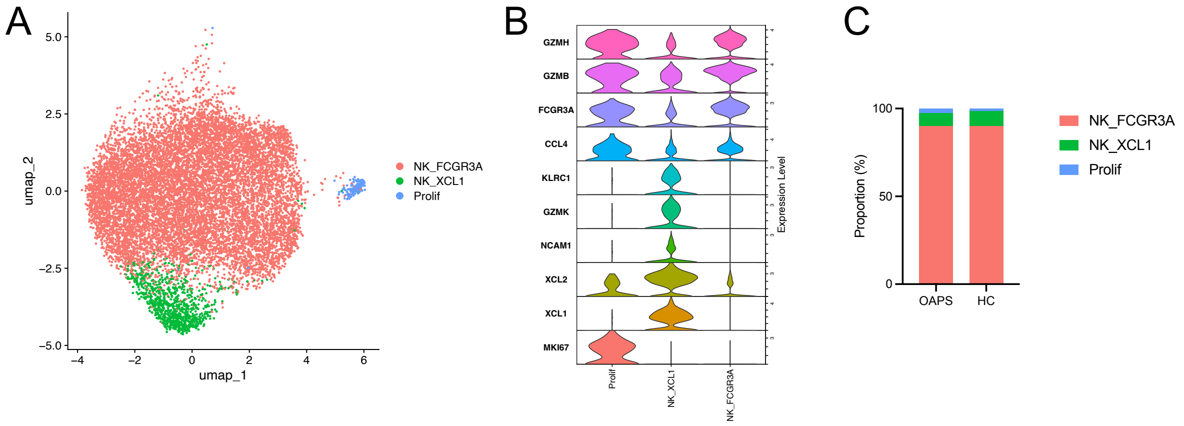

Supplementary Figure 7. The subclusters of NK cells in PBMCs.

(A) UMAP of NK subclusters in PBMCs. Different colors mean different subclusters. (B) Violin plot showing the expressions of marker genes in NK subclusters. (C) Bar plot showing the proportions of NK subclusters in PBMCs from OAPS patients and HCs.

OAPS, obstetric antiphospholipid syndrome; HCs, healthy controls; PBMC, peripheral blood mononuclear cells; NK, natural killer cell.

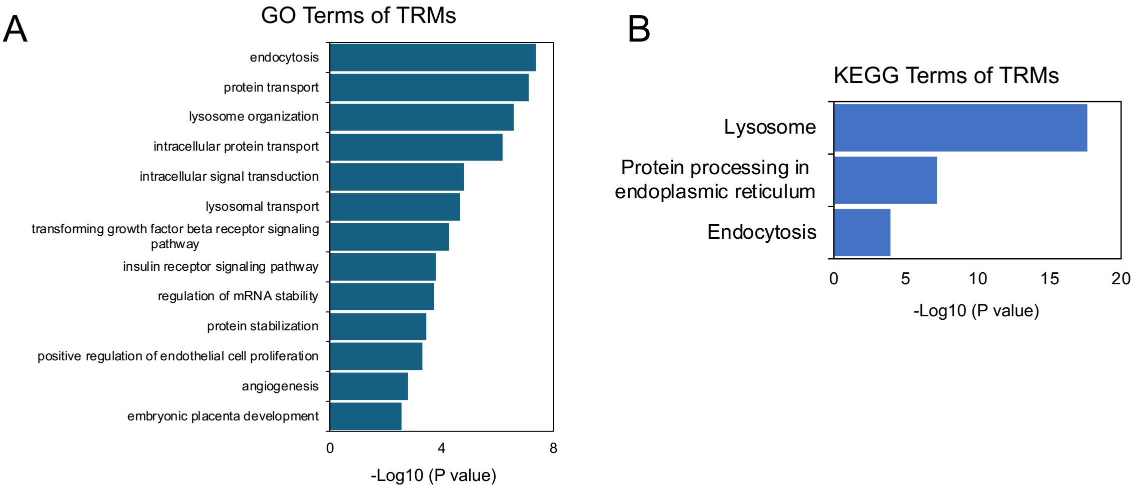

Supplementary Figure 8. Functions of decidual TRMs.

(E) Bar plot showing the GO terms of marker genes in decidual TRMs. (F) Bar plot showing the KEGG terms of marker genes in decidual TRMs.

TRMs, tissue-resident macrophages.

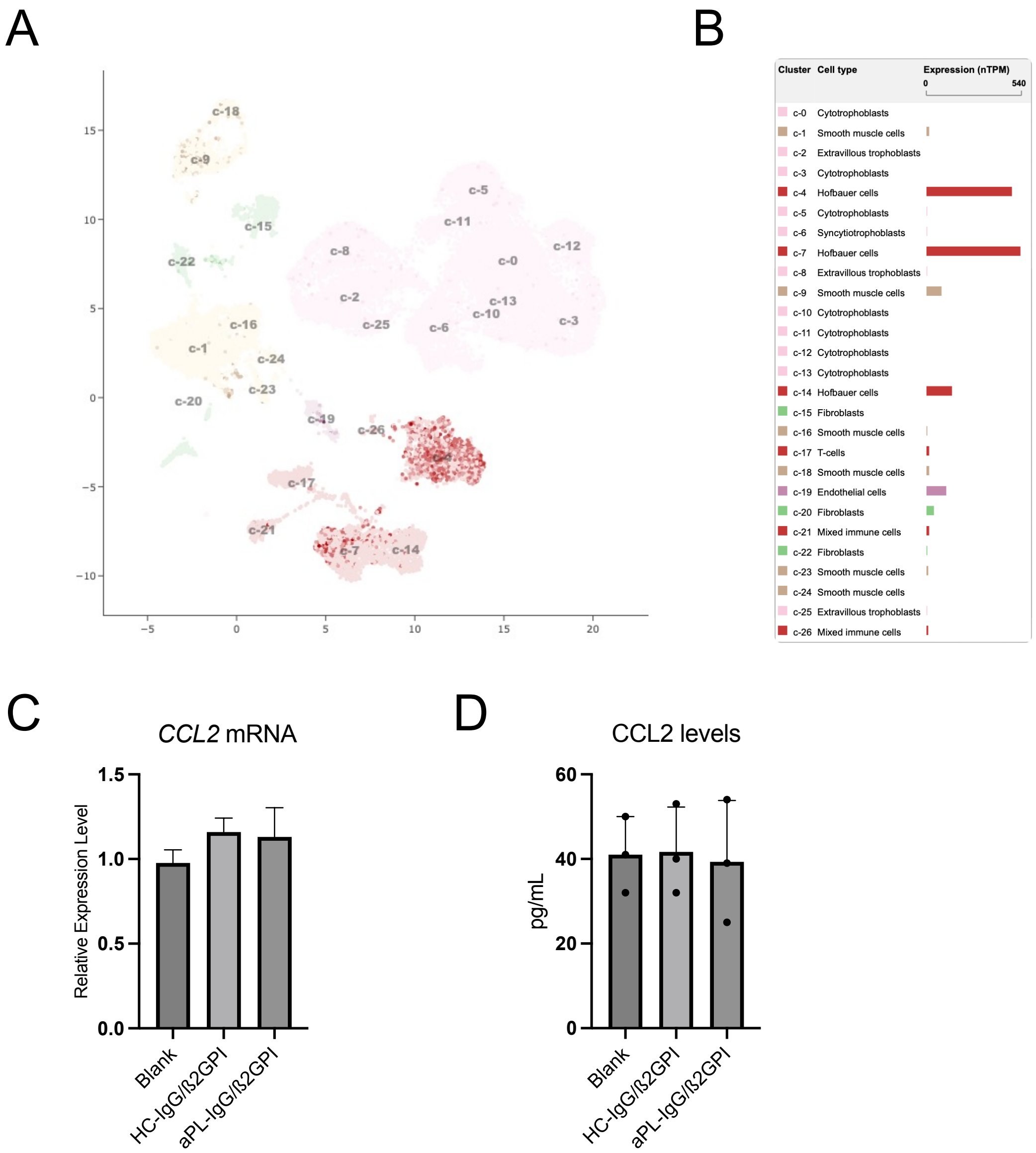

Supplementary Figure 9. The expression of CCL2 in trophoblasts.

(A) UMAP visualization of cell clusters in a placental scRNA-seq dataset obtained from the Human Protein Atlas database (https://www.proteinatlas.org). (B) Table showing *CCL2* gene expression levels in different cell clusters of placental tissue. (C) Bar plot showing the effect of aPL-IgG/β2GPI complex on *CCL2* gene expression in HTR-8/SVneo cells, as determined by RT-qPCR (n=3). (D) Bar plot showing the effect of aPL-IgG/β2GPI complex on CCL2 levels in culture supernatant in HTR-8/SVneo cells, as measured by ELISA (n=3). scRNA-seq, single cell RNA sequencing; aPL, antiphospholipid antibody; Ig, immunoglobin; β2GPI, β 2 glycoprotein I; RT-qPCR, reverse transcription quantitative polymerase chain reaction; ELISA, enzyme-linked immunosorbent assay.

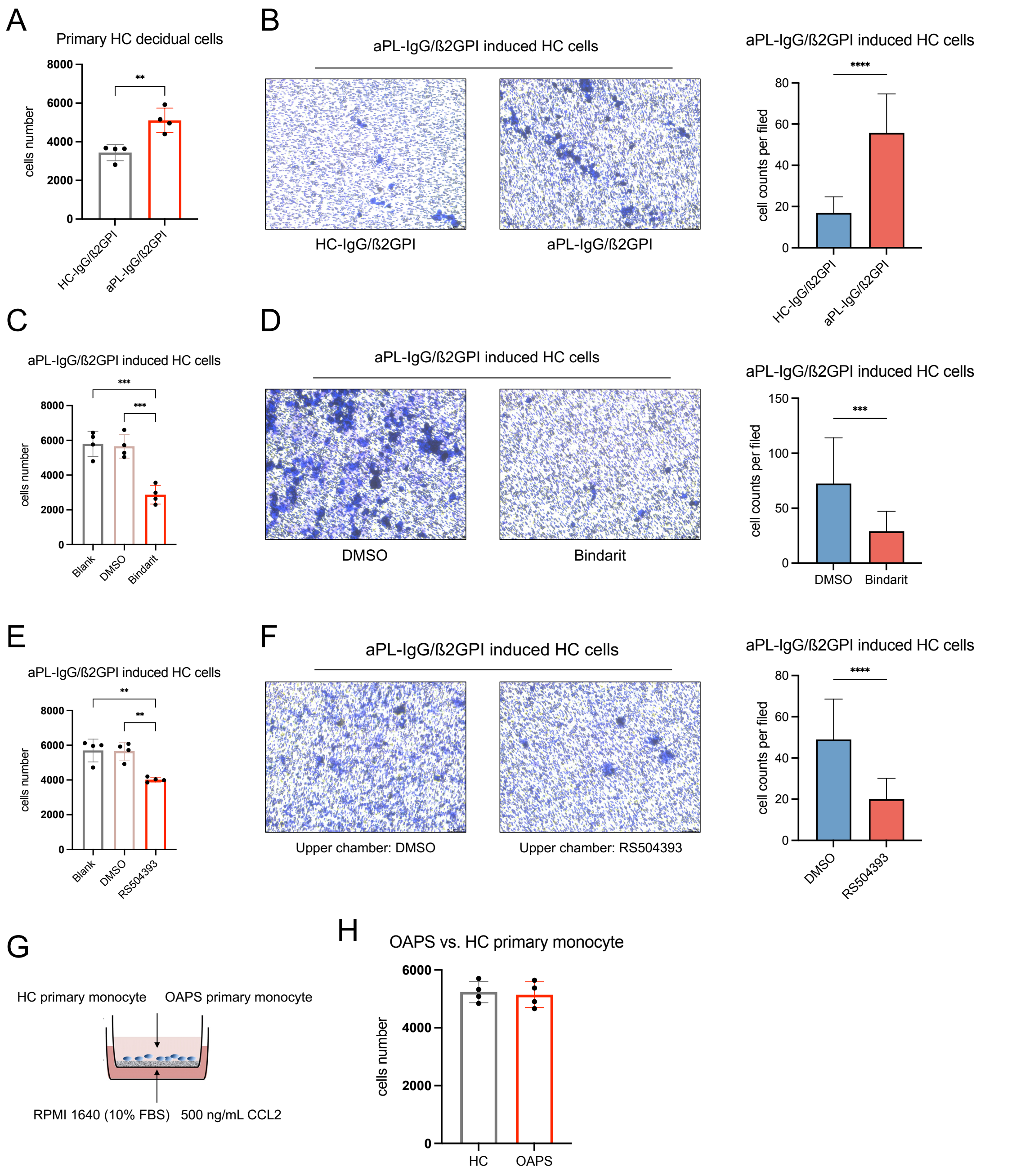

Supplementary Figure 10. The chemotaxis from aPL-IgG/β2GPI complex-induced HCs primary decidual cells to THP-1 and THP-1 M0 in vitro.

(A) Bar plot showing the numbers of THP-1 in the down chamber after chemotaxis by decidual cells of HCs stimulated by HC-IgG/β2GPI and aPL-IgG/β2GPI complex (n=4). (B) Representative images and statistical results of THP-1 M0 macrophages passed the Transwell membrane (n=4). (C) Bar plot showing the numbers of THP-1 in the down chamber after chemotaxis by decidual cells of HCs stimulated by aPL-IgG/β2GPI complex with or without Bindarit treatment (n=4). (D) Representative images and statistical results of THP-1 M0 macrophages passed the Transwell membrane (n=4). (E) Bar plot showing the numbers of

THP-1 with or without RS504393 treatment in the down chamber after chemotaxis by decidual cells of HCs stimulated by aPL-IgG/β2GPI complex (n=4). (F) Representative images and statistical results of THP-1 M0 macrophages passed the Transwell membrane (n=4). (G) Schematic diagram of an in-vitro chemotaxis system where CCL2 chemoattracting peripheral monocytes extracted from HCs and OAPS patients. (H) Bar plot showing the numbers of monocytes in the down chamber after chemotaxis by recombinant CCL2 (n=4). For A, B, D, F and H, data are shown as mean ± SD and were analyzed by Students' t-test. For C and E, data are shown as mean ± SD and were analyzed by One-way ANOVA. The Dunnett test was used for multiple comparisons. **p<0.001, ***P<0.001, ****<P<0.0001. OAPS, obstetric antiphospholipid syndrome; HCs, healthy controls; aPL, antiphospholipid antibody; Ig, immunoglobin; β2GPI, β2 glycoprotein I.

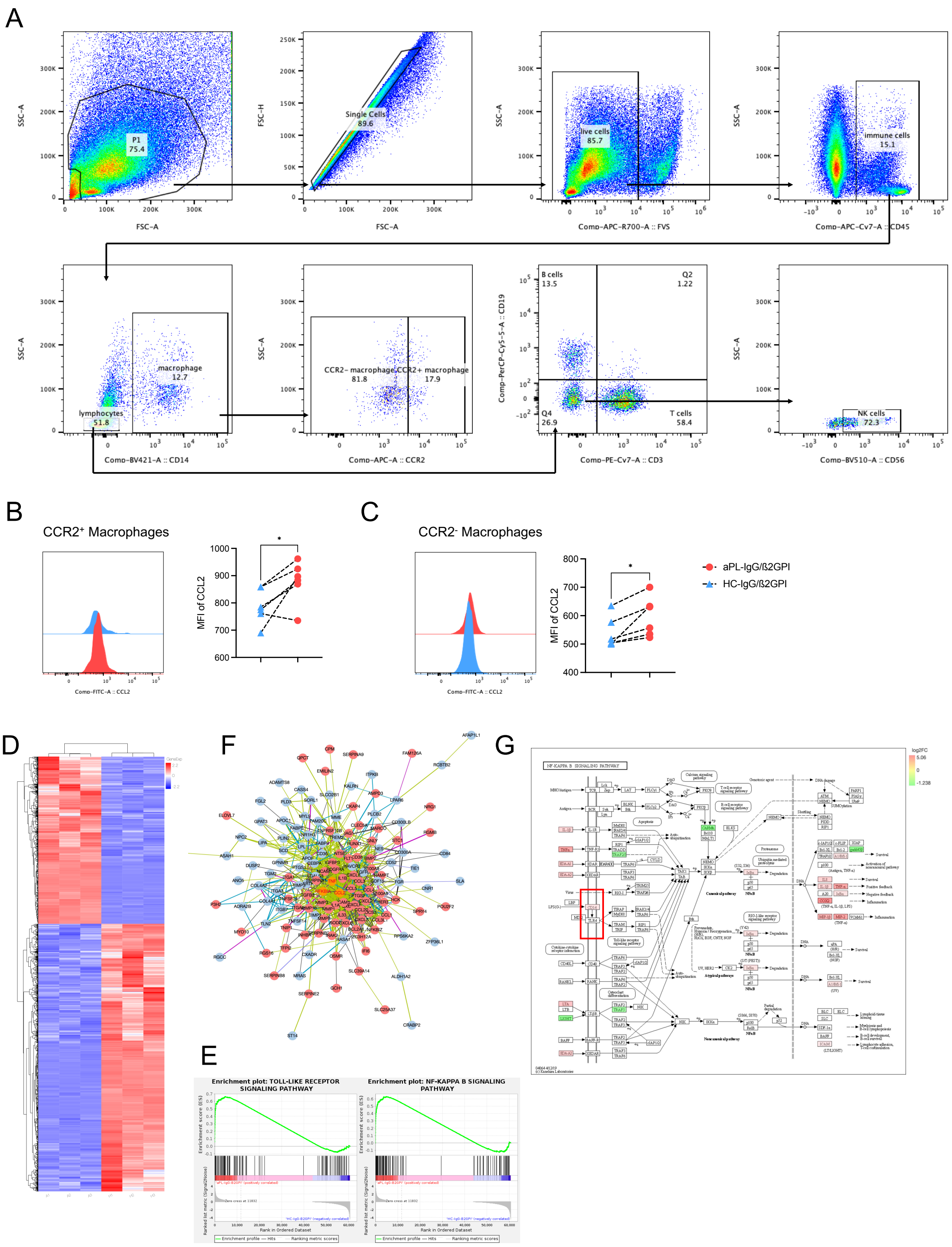

Supplementary Figure 11. The source and mechanism of CCL2 production in OAPS decidua.

(A) Gating strategy for flow cytometry that exploring the source of CCL2 in decidua. FCM analysis of CCL2 secretion in decidual CCR2^{+} macrophages (B) and CCR2^{-} macrophages (C) from HCs' decidua after stimulation with HC-IgG/β2GPI and aPL-IgG/β2GPI complexes. Data are presented as mean ± SD and were analyzed using a paired t-test. *P < 0.05. (D) Heatmap showing the mRNA expression profiles in THP-1 M0 macrophages after stimulation

with HC-IgG/β2GPI and aPL-IgG/β2GPI complexes. (E) GESA plot showing that the TLR signaling pathway and NF-κB pathway were enriched in THP-1 M0 after stimulated by aPL-IgG/β2GPI complex. (F) Protein-protein interaction network of the DEGs in THP-1 M0 after stimulated by HC-IgG/β2GPI and aPL-IgG/β2GPI complex by STRING database. (G) KEGG pathway plot showing the DGEs in Toll-like receptor mediated pathway.

OAPS, obstetric antiphospholipid syndrome; HCs, healthy controls; aPL, antiphospholipid antibody; Ig, immunoglobin; β2GPI, β2 glycoprotein I; FCM, flow cytometry; GSEA, gene set enrichment analysis.

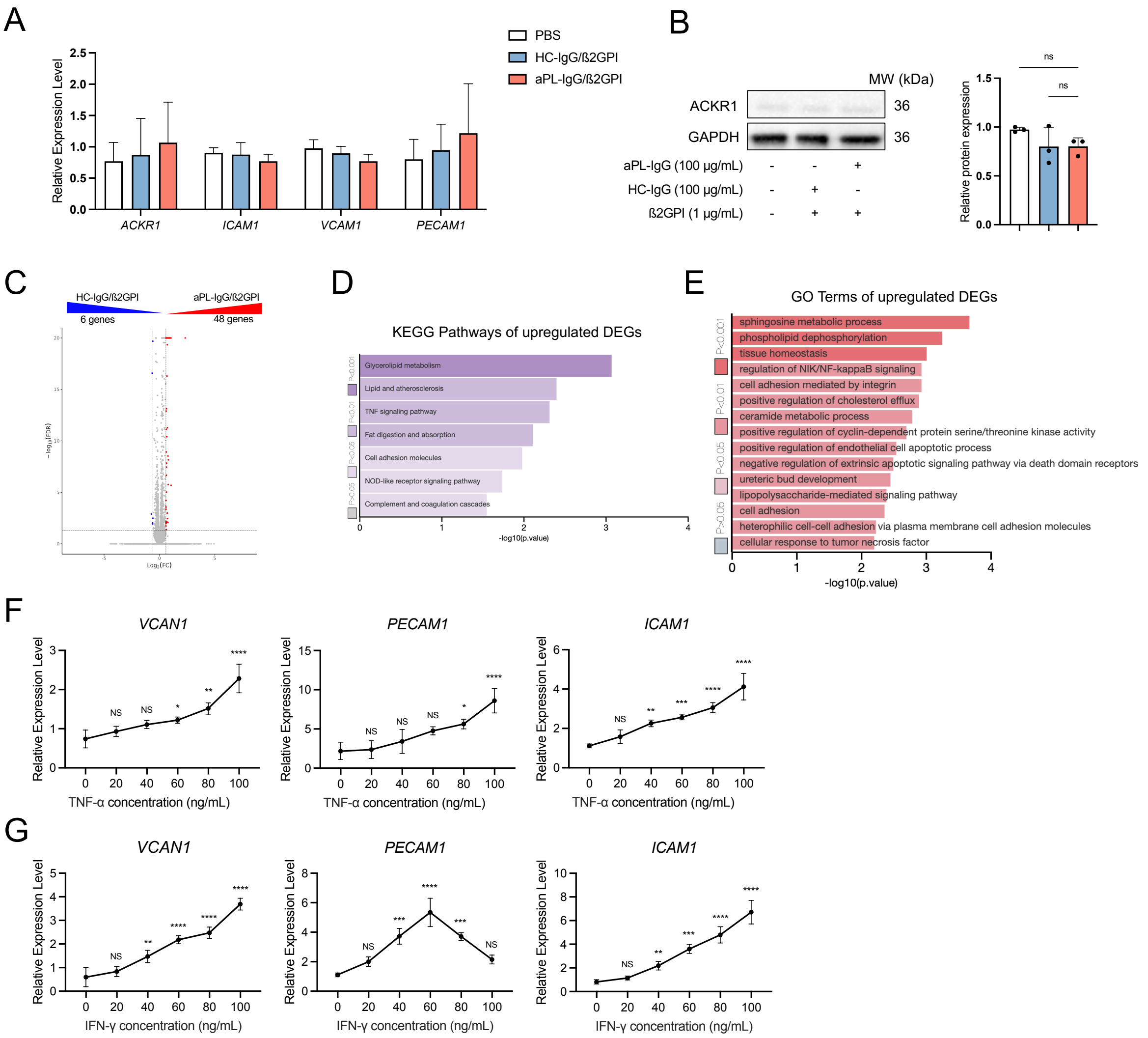

Supplementary Figure 12. The effects of aPL-IgG/β2GPI complex on EA.hy926 cells in vitro.

(A) Bar plot showing the expressions of *ACKR1*, *ICAM1*, *VCAM1* and *PECAM1* genes in EA.hy926 cells under the stimulation of PBS, HC-IgG/β2GPI and aPL-IgG/β2GPI complex. (B) Representative immunoblot and semi-quantified results of ACKR1 protein expression in

EA.hy926 cells under different stimulations (n=3). (C) Volcano plot showing the DEGs of EA.hy926 cells between the stimulation of HC-IgG/β2GPI and aPL-IgG/β2GPI complex. (D) Enriched KEGG terms of upregulated DEGs in EA.hy926 cells stimulated by aPL-IgG/β2GPI complex. (E) Enriched GO terms of upregulated DEGs in EA.hy926 cells stimulated by aPL-IgG/β2GPI complex. (F) Line chart showing the expression of *VCAN1, PECAM1 and ICAM1* genes in EA.hy926 cells under different concentrations of TNF-α (n=3). (G) Line chart showing the expression of *VCAN1, PECAM1 and ICAM1* genes in EA.hy926 cells under different concentrations of IFN-γ (n=3).

Data are shown as mean ± SD and were analyzed by One-way ANOVA. The Dunnett test was used for multiple comparisons. *P<0.05, **p<0.001, ***P<0.001, ****<P<0.0001.

OAPS, obstetric antiphospholipid syndrome; HCs, healthy controls; aPL, antiphospholipid antibody; Ig, immunoglobin; β2GPI, β2 glycoprotein I; DEGs, differential expressed genes.

Supplementary Tables

Supplementary Table 1. Clinical information of participants whose PBMCs were used for experiments.

	OAPS group (n=30)	HC group (n=17)
Age (years)	28.8±4.4	30.9±3.3
Gestation (weeks)		
5+0 ~ 6+6	14 (46.7)	8 (47.1)
7+0 ~ 8+6	7 (23.3)	6 (35.3)
9+0 ~ 10+6	9 (30.0)	3 (17.6)
LA1/LA2 > 1.2 [n (%)]	26 (86.7)	-
aCL Ab IgM positive [n (%)]	17 (56.7)	-
aCL Ab IgG positive [n (%)]	5 (16.7)	-
aβ2GPI Ab IgM positive [n (%)]	8 (26.7)	-
aβ2GPI Ab IgG positive [n (%)]	6 (20.0)	-
Miscarriage < 16 weeks [n (%)]		
1~2 times	18 (60.0)	0 (0)
≥ 3 times	11 (36.7)	0 (0)
Stillbirth ≥ 16 weeks [n (%)]	15 (50.0)	0 (0)
Severe pre-eclampsia [n (%)]	7 (23.3)	0 (0)
Severe PI [n (%)]	5 (16.7)	0 (0)
Premature delivery < 34 weeks [n (%)]	0 (0)	0 (0)
Thrombosis [n (%)]	1 (3.3)	0 (0)
Embryo demise [n (%)]	7 (23.3)	0 (0)
Treatment [n (%)]		
LDA	30 (100.0)	0 (0)
LMWH	21 (70.0)	0 (0)
Progesterone	14 (46.7)	0 (0)

Footnotes: LA, lupus anticoagulant; aCL, anticardiolipin antibody; aβ2GPI, anti β2 glycoprotein I antibody; PI, placental insufficiency; LDA, low dosage of aspirin; LMWH, low molecular weight heparin; PBMCs, peripheral blood mononuclear cells.

Supplementary Table 2. Clinical information of participants whose decidua was used for experiments.

	OAPS group (n=11)	HC group (n=21)
Age (years)	31.3±2.4	32.1±3.0
Gestation (weeks)		
6+0 ~ 6+6	0 (0)	6 (28.6)
7+0 ~ 7+6	1 (9.1)	6 (28.6)
8+0 ~ 8+6	3 (27.3)	8 (38.1)
9+0 ~ 9+6	7 (63.6)	1 (4.8)
LA1/LA2 > 1.2 [n (%)]	9 (81.8)	-
aCL Ab IgM positive [n (%)]	6 (54.5)	-
aCL Ab IgG positive [n (%)]	4 (36.4)	-
aβ2GPI Ab IgM positive [n (%)]	1 (9.1)	-
aβ2GPI Ab IgG positive [n (%)]	2 (18.2)	-
Miscarriage < 16 weeks [n (%)]		
1~2 times	4 (36.4)	0 (0)
≥ 3 times	7 (63.6)	0 (0)
Stillbirth ≥ 16 weeks [n (%)]	3 (27.3)	0 (0)
Severe pre-eclampsia [n (%)]	3 (27.3)	0 (0)
Severe PI [n (%)]	6 (54.5)	0 (0)
Premature delivery < 34 weeks [n (%)]	1 (9.1)	0 (0)
Thrombosis [n (%)]	3 (27.3)	0 (0)
Treatment [n (%)]		
LDA	11 (100)	0 (0)
LMWH	9 (81.8)	0 (0)
Immunosuppressive agent	0 (0)	0 (0)
Progesterone	4 (36.4)	0 (0)

Footnotes: LA, lupus anticoagulant; aCL, anticardiolipin antibody; aβ2GPI, anti β2 glycoprotein I antibody; PI, placental insufficiency; LDA, low dosage of aspirin; LMWH, low molecular weight heparin.

Supplementary Table 3. Clinical information of participants whose serum was used for extraction of polyclonal IgG.

Sample No.	Age (years)	Gestation (weeks)	aβ2GPI-IgG (IU/L)	aCL-IgG (IU/L)
OAPS31	31	32+4	70.23	80.95
OAPS32	29	16+4	117.20	80.22
OAPS33	31	-	149.43	130.74
OAPS34	27	24+1	76.85	22.82
OAPS35	38	7+5	>500	384.49
OAPS36	30	16+4	267.62	85.49

Footnotes: LA, lupus anticoagulant; aCL, anticardiolipin antibody; aβ2GPI, anti β2 glycoprotein I antibody; PI, placental insufficiency; LDA, low dosage of aspirin; LMWH, low molecular weight heparin. aβ2GPI-IgG and aCL-IgG were detected by chemiluminescent immunoassay, 40 IU/L is the cut-off value of high-titers positive.
